# Targeted nanoparticles for placenta-specific drug delivery in pregnant rhesus macaques

**DOI:** 10.7150/thno.115081

**Published:** 2026-01-01

**Authors:** Jenna K. Schmidt, Ann M. Mitzey, Logan T. Keding, Sarah A. Shaw, Lewis Renshall, Frances Beards, Mona H. Al-Mugotir, Heather A. Simmons, Puja Basu, Michele L. Schotzko, Emily Ren, Thaddeus G. Golos, Lynda K. Harris

**Affiliations:** 1 Wisconsin National Primate Research Center, University of Wisconsin-Madison, Madison, WI, USA.; 2 Department of Comparative Biosciences, School of Veterinary Medicine, University of Wisconsin-Madison, WI, USA.; 3 Department of Obstetrics and Gynecology, University of Wisconsin-Madison, University of Wisconsin-Madison, WI, USA.; 4 Maternal and Fetal Health Research Centre, Division of Developmental Biology and Medicine, The University of Manchester, Manchester, UK.; 5 St. Mary’s Hospital, Manchester University NHS Foundation Trust, Manchester Academic Health Science Centre, Manchester, UK.; 6 Olson Center for Women’s Health, Department of Obstetrics and Gynecology, University of Nebraska Medical Center, Omaha, NE, USA.

**Keywords:** liposome, placenta, pregnancy, drug delivery, targeting.

## Abstract

**Rationale:** To reduce the risks associated with systemic drug administration in pregnancy and create new therapies for placental dysfunction, we have previously developed targeted liposomes capable of selective delivery of payloads to the placentas of pregnant mice. In this study, we assessed whether these liposomes selectively accumulated in the placentas of nonhuman primates and evaluated the maternal systemic response to liposome infusion.

**Methods:** Liposomes were produced using the thin-film method, decorated with the placental homing peptide CRGDKGPDC (iRGD) or non-targeting peptide ARALPSQRSR (ARA) and loaded with 5(6)carboxyfluorescein. Liposomes were infused intravenously into pregnant rhesus macaques early in gestation, at days 45-64 (iRGD, n = 4; ARA, n = 1) or mid-gestation, at days 84-100 (iRGD, n = 4; ARA, n = 2; term = 165 days). After 24 h, maternal blood and maternal, uteroplacental and fetal tissues were collected for evaluation.

**Results:** Liposome infusions were well tolerated, with no adverse clinical reactions or abnormal placental or fetal pathology. Maternal plasma hormone, cytokine and chemokine levels, and complete blood cell counts remained broadly stable for the duration of the treatment period. iRGD-decorated liposomes accumulated in both placental discs of each animal, varying in intensity and tissue distribution. Limited fluorescence was noted in some sections of maternal liver, spleen, kidney and mesenteric lymph nodes, but no visible signal was observed in other maternal or fetal tissues examined. In contrast, ARA-decorated liposomes exhibited a widespread tissue distribution, with fluorescence observed in the majority of maternal, uteroplacental and fetal tissues examined.

**Conclusions:** This study confirms that iRGD-decorated liposomes selectively accumulate in the rhesus macaque placenta *in vivo*, but are not transferred to the fetus. Their composition is compatible with short term courses of treatment, indicating that this nanoparticle formulation may be suitable for targeted placental delivery of therapeutic payloads in human pregnancy.

## Introduction

Appropriate placentation is critical for a successful pregnancy in all viviparous mammals, as it ensures that an adequate supply of nutrients and oxygen reach the developing fetus. Impaired placental development and function are strongly associated with adverse pregnancy outcomes, including miscarriage, pre-eclampsia (PE), fetal growth restriction (FGR) and stillbirth 1. In addition, deficits in placental structure and/or function expose the fetus to an adverse environment in utero, with the compensatory fetal adaptations to hypoxia or undernutrition causing developmentally programmed life-long health complications 2-5. It is therefore critical to develop interventions to treat placental dysfunction, reducing the incidence and severity of pregnancy complications and improving the short- and long-term health of pregnant individuals and their offspring.

Humans, monkeys and great apes possess a hemochorial placenta, which is characterized by direct contact between the maternal blood and the fetal-derived placental tissue. Placentas in these species are villous in structure, with each villus consisting of an outer layer of syncytiotrophoblast, an underlying layer of proliferative cytotrophoblasts and a core of villous stroma, fetal blood vessels, and specialized macrophages called Hofbauer cells 1. In early pregnancy, a population of invasive extravillous trophoblasts (EVT) migrate from the tips of placenta villi into the uterine wall, anchoring the placenta and colonizing the maternal decidua, myometrium and uterine spiral arteries. In healthy pregnancy, EVT remodel the uterine spiral arteries in the decidua and proximal third of the myometrium, directly replacing the endothelium and vascular smooth muscle cells to create dilated channels which lack the ability to contract 6, 7. Incomplete remodeling of the uterine spiral arteries leads to impaired delivery of blood to the placenta, local oxidative and nitrative stress, damage to the syncytiotrophoblast layer, and increased shedding of placental material into the maternal circulation. Reduced uteroplacental perfusion can also impair transfer of oxygen and nutrients across the placenta, directly contributing to reduced fetal growth. In addition, other phenotypes of placental dysfunction contribute to adverse pregnancy outcomes in PE and FGR, including reduced expression and activity of nutrient transporters, elevated rates of trophoblast cell death and activation of inflammatory pathways 8, 9.

Suboptimal fetal growth and impaired uteroplacental perfusion can be diagnosed by Doppler ultrasound; however, there are currently no available interventions to treat these conditions 10, 11. Sustained underfunding of maternal and perinatal health research 12, the actual and perceived risks associated with drug testing in pregnancy, and a lack of commercial investment in obstetric drug development 13, has led to this situation. To address this, our lab has developed methods for selectively targeting therapeutics to the placenta and the maternofetal interface (MFI), to provide safer and more effective means of treating pregnancy complications. This approach reduces the likelihood of detrimental off-target effects on maternal physiology and minimizes drug transfer to the fetus, as well as increasing drug efficacy in comparison to systemic administration.

The concept of peptide-mediated tissue targeting is based on the principle that the vascular endothelium of each organ expresses one or more unique cell surface molecules or “vascular zip codes”, and that it is possible to identify random “homing peptide” sequences that bind to these unique, tissue-specific zip codes 14. When administered intravenously, the homing peptides only come into contact with the endothelial cells lining the vasculature of each organ, although in pregnancy they will also encounter the outer syncytiotrophoblast layer of the human placenta, which is directly bathed in maternal blood. Thus, it is the endothelial (or placental) cell surface localization of the peptide receptors which determines the tissue specificity of peptide homing: even if that same receptor molecule is expressed by another cell type, intravenously administered peptides are confined to the vascular network and will never come into contact with them, preventing off-target binding and accumulation. Similarly, intravenously administered nanoparticles decorated with tissue-specific homing peptides are confined to the vasculature and will only be present in the vascular beds of tissues or organs expressing the cognate receptor for that peptide. We have previously identified a number of uteroplacental homing peptides, including the sequences CRGDKGPDC (iRGD) and NKGLRNK (NKG), and used these to create targeted liposomes for placenta-specific drug delivery. We have validated the safety and efficacy of these formulations *in vivo*, in mouse models of placental dysfunction and ex vivo using human placental explant cultures 15, 16. We have demonstrated placenta-specific targeting using fluorescent payloads and shown that placenta-specific drug delivery can improve fetal growth and placental function 16. For example, we have shown that targeted delivery of insulin-like growth factor-II (IGF-II) to the placenta increased fetal and placental weights in a mouse model of FGR 16, and targeted delivery of a nitric oxide donor to the endothelium of the maternal uterine spiral arteries normalized fetal weight and spiral artery diameter in endothelial nitric oxide synthase knockout (eNOS^-/-^) mice 15. We have also demonstrated that targeted delivery of epidermal growth factor (EGF) to human placental explants improves amino acid transporter activity 17.

Our overall therapeutic goal is to create tunable, targeted liposomes to selectively courier a range of small molecule therapeutics directly to the MFI, to correct the diverse phenotypes of placental dysfunction observed in human pregnancy. These include delivery of vasodilators to improve uteroplacental blood flow, antioxidants to mitigate oxidative damage, growth factors to increase placental volume, surface area and nutrient transport, and anti-inflammatory agents to reduce local inflammation. Due to differences in structure, and in some regards (endocrine, immune) function of the placenta across species, there are limitations to the translatability of results from mouse models to humans. Given the importance of obtaining robust safety data prior to embarking on clinical trials in pregnant humans, it is imperative to seek more physiologically relevant models in which to test new interventions. Nonhuman primates have the most similar anatomy and physiology to humans and thus, represent the most clinically relevant mammalian model for experimental therapeutic approaches. The objectives of this study were to determine whether liposomes decorated with the iRGD peptide sequence selectively target the nonhuman primate MFI and to assess the short-term effects of liposome administration on maternal physiology and placental histology.

## Methods

### Ethics statement

The nonhuman primates used in this study were cared for by the staff at the Wisconsin National Primate Research Center (WNPRC) in accordance with the regulations and guidelines outlined in the Animal Welfare Act and the Guide for the Care and Use of Laboratory Animals and the recommendations of the Weatherall report 18. This study was approved by the University of Wisconsin-Madison Graduate School Institutional Animal Care and Use Committee (protocol number g006040). While on study, all animals were evaluated by trained animal care staff at least twice daily for signs of pain, distress, and illness by observing appetite, stool quality, activity level, and physical condition. Animals exhibiting abnormal presentation for any of these clinical parameters were provided appropriate care by the veterinarians.

### Animals

Initial experiments were conducted to assess the effectiveness of the route of delivery. iRGD-decorated liposomes were administered to mid-gestation pregnant females by ultrasound-guided intraplacental injection into one cynomolgus macaque (*Macaca fascicularis*) and one rhesus macaque (*Macaca mulatta*) and by intravenous infusion via the saphenous vein into one common marmoset (*Callithrix jacchus*) and one cynomolgus macaque.

**Figure 1:**
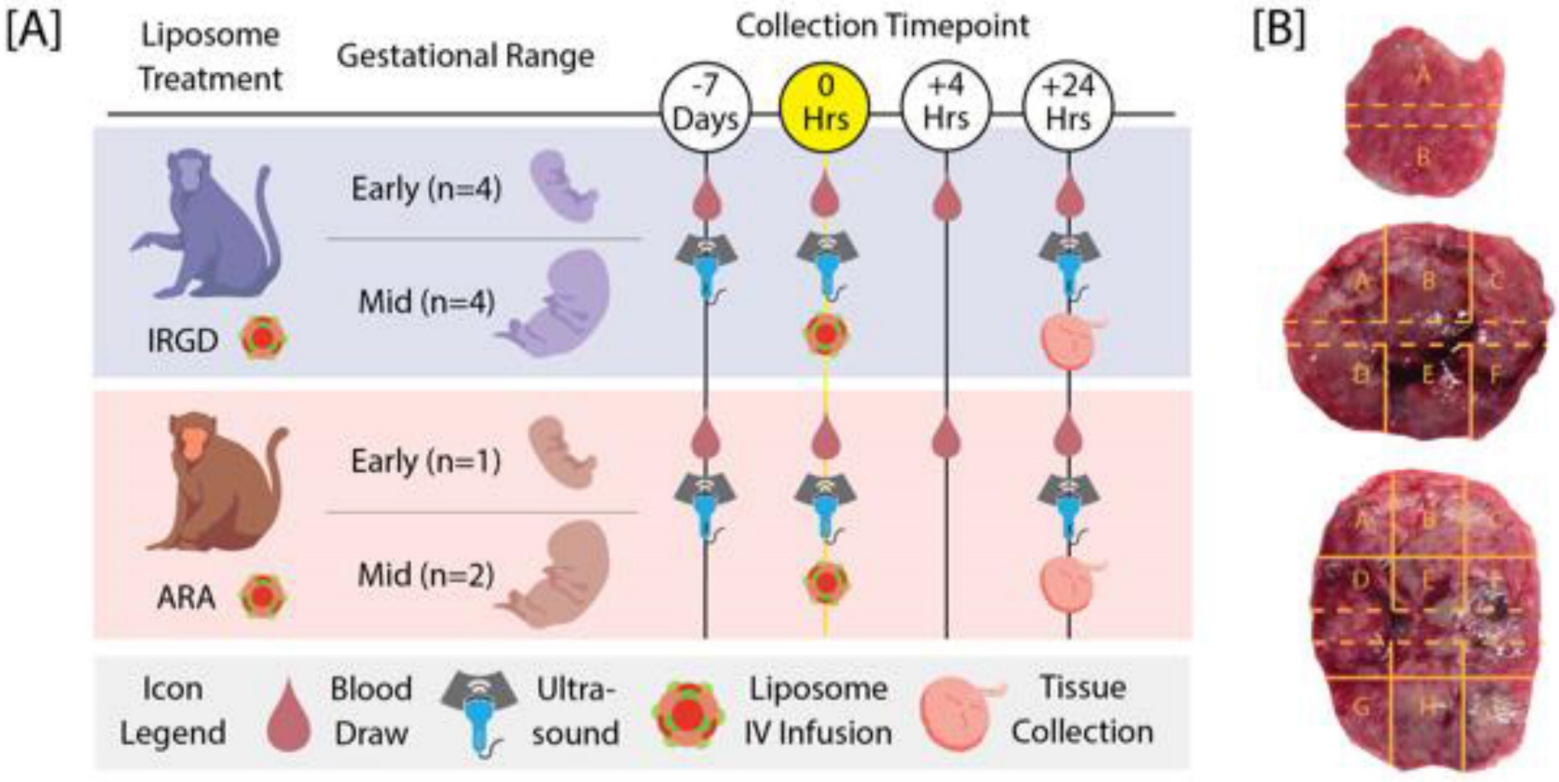
** Overview of the experimental design and placental tissue processing. [A]** Timeline of procedures for rhesus macaques that underwent intravenous infusion of iRGD or ARA-decorated liposomes during early or mid-pregnancy. Blood draws and ultrasounds were performed to assess maternal response to infusion and tissues were collected 24 h post-infusion. **[B]** Placenta tissue was dissected into 2-6 or 9 regions following a grid pattern for early and mid-gestation, respectively, to assess liposome targeting. The region within the dashed lines indicates the placental center cut collected for histopathological analysis.

After these pilot experiments, liposomes were administered by intravenous infusion to nine additional pregnant rhesus macaques (*Macaca mulatta*). The rhesus macaques enrolled in this study were between 4-19 years old and weighed between 5.2-11.8 kg. One female was enrolled in three experimental groups in three different pregnancies. The female macaques were pair-housed with compatible males following the observation of menses. Pregnancy was detected by abdominal ultrasound and gestational age was estimated as previously described 19. Pregnant macaques received iRGD liposomes at either gestational day (GD) 45-64 (n = 4) (referred to hereafter as early gestation), or GD 84-95 (n = 4) (referred to hereafter as mid-gestation) or received control ARA-decorated liposomes at early (n = 1) or mid-gestation (n = 2). An overview of the experimental design is provided in Figure 1A.

### Liposome preparation

Liposomes were prepared using the thin film method from 1,2-distearoyl-*sn*-glycero-3-phospho-choline (DSPC; 32.5 mM), 1,2-distearoyl-*sn*-glycero-3-phosphoethanolamine-*N*-[methoxy(polyethylene glycol)-2000] ammonium salt (DSPE-PEG(2000); 1.875 mM), 1,2-distearoyl-*sn*-glycero-3-phosphoethanola-mine-*N*-[maleimide(polyethyleneglycol)-2000] ammonium salt (DSPE-PEG(2000)-maleimide; 0.625 mM; Avanti Polar Lipids), and cholesterol (15 mM; Sigma-Aldrich), as previously described 16. Lipids were dissolved in chloroform (Sigma-Aldrich), which was then removed by rotary evaporation (40 °C, 270 mbar) to create a thin lipid film. The film was dried in a vacuum oven overnight at room temperature and rehydrated with sterile PBS containing 5(6)-carboxyfluorescein (FAM, 2.5 mM; Sigma-Aldrich). The resulting suspension was warmed to 55 °C in a shaking incubator and manually vortexed every 15 min for a minimum of 2 h. The suspension was extruded using a thermobarrel Mini-Extruder and 200 nm-pore polycarbonate membranes (Avanti Polar Lipids) a minimum of 11 times to produce a monodisperse liposome formulation containing encapsulated FAM.

To facilitate surface decoration, liposomes were incubated with the placental homing peptide rhodamine-CCRGDKGPDC (iRGD; 1.25 μM; Insight Biotechnology) or the control peptide rhodamine-CARALPSQRSR (ARA; 1.25 μM; Insight Biotechnology) overnight at room temperature to enable conjugation of free thiol groups on the N-terminal cysteine residues to maleimide groups on the liposomal surface via a Michael-type addition reaction. Unbound peptide and unencapsulated FAM were removed by dialysis against sterile PBS for 24 h. Liposomes were stored at 4 °C and Z-average sizes and polydispersity indices (PDI) were calculated from the size distributions measured by dynamic light scattering at 25 °C (Zetasizer Nano). Each liposome formulation was submitted to the University of Wisconsin Veterinary Care Diagnostic Pathology Services Laboratory to confirm sterility prior to administration.

### Liposome delivery to pregnant rhesus macaques

Pregnant macaques were anesthetized with an intramuscular dose of ketamine (~10 mg/kg) and an abdominal ultrasound was performed prior to, and after liposome administration to monitor fetal health. Up to 5 mg/kg of diphenhydramine was administered approximately 30 min prior to liposome delivery. Liposomes were delivered intravenously via the saphenous vein, beginning at an infusion rate of 1 mL/min and increasing the rate to 5 mL/min after 5 min. The total infusion volume was calculated to be approximately 5% (v/v) of the animals’ total blood volume which equated to approximately 15 – 33 mL of liposome suspension, with a mean liposome content of 1.82 X 10^13^ particles per mL. One animal, e11, received iRGD-decorated liposomes containing PBS instead of FAM. Animals were monitored by pulse oximeter during the infusion and after the infusion were monitored closely for any apparent adverse clinical symptoms (i.e., changes in complete blood counts (CBCs), fever, appetite and activity).

### Collection of blood and tissues

Maternal blood samples were collected a week prior to treatment, on the day of treatment just prior to infusion, and at 4 h and 24 h post-treatment. Blood samples were obtained from the saphenous vein using a vacutainer system. CBCs were performed as previously described 20. Serum and plasma were isolated by centrifugation at 1300 x *g* for 10 min at room temperature, and then aliquoted and stored at -80 ºC for subsequent analysis of hormones and immunomodulatory proteins in maternal circulation.

The conceptus (i.e., fetus, placenta, and fetal membranes) was surgically collected at 24 h post-treatment via caesarean section and fetectomy. Fetuses were euthanized by intravenous or intracardiac injection of at least 50 mg/kg sodium pentobarbital. Maternal tissue biopsies of liver, kidney, lymph node and uterine placental bed were collected aseptically and then the animals were recovered from anesthesia. All the animals used for the pilot experiments (one marmoset, two cynomolgus macaques, one rhesus macaque) were euthanized immediately prior to tissue collection to evaluate maternal tissues. Cardiac perfusion with heparinized saline was performed to remove the blood from tissues and any circulating liposomes prior to tissue harvest. Maternal and fetal tissues were processed for histopathological analysis, and fresh flash-frozen tissues were processed for fluorescent microscopy. A center cut of the primary placental disc containing the umbilicus and the secondary placental disc were collected for histopathological analysis and the remaining placental disc tissue was dissected into 2-6 or 9 regions following a grid pattern for early and mid-gestation, respectively, and the tissue was flash frozen (Figure 1B).

### Histopathology

Tissue specimens were fixed in 4% (w/v) paraformaldehyde, dehydrated in ethanol, paraffin embedded, sectioned and stained with hematoxylin and eosin. Histological evaluations of fetal, maternal, and MFI tissues were performed by board-certified veterinary pathologists (HAS, PB) blinded to the treatment. Histology of treated subjects was compared to archived non-treated gestational age-matched maternal, fetal, and MFI samples including placenta (n = 3 GD 44-45; n = 8 GD 100-110).

### Analysis of liposome targeting in tissues

To detect the signals from the rhodamine-labeled peptides on the liposomal surface and the FAM cargo, tissue specimens were flash frozen in liquid nitrogen, embedded in OCT and stored at -80 °C until sectioned at 5-10 µm on a Leica CM3050 S cryostat. Every tenth section of each tissue block was transferred to a glass microscope slide, fixed in ice-cold methanol, washed in PBS (3 X 5 min), mounted with Vectashield mounting medium containing DAPI and imaged with a Nikon ECLIPSE-Ti2 microscope. Tissues were then reviewed for presence or absence of a fluorescent signal, noting the localization. Maternal and fetal organs (n = 8-25 sections per organ biopsy per animal) and placental discs (n = 52–128 sections per biopsy per animal) were analyzed. To calculate the percentage of positive tissue sections per organ biopsy shown in Figure 4, the number of tissue sections showing evidence of a fluorescent signal was divided by the total number of tissue sections imaged of that biopsy and multiplied by 100. To prepare the fluorescence distribution map in Figure 4D, these values were rounded to the nearest 5%.

### Hormone assays

For the rhesus macaques that received intravenous delivery of liposomes, maternal serum levels of estradiol (E2) and progesterone (P4) were measured by the WNPRC Assay Services Unit using established methods 21. Briefly, steroid hormones were extracted from a 400 µL aliquot of serum and evaluated for E2 and P4 levels using a Roche Cobas e411 analyzer equipped with ElectroChemi-Luminescence technology according to manufacturer instructions.

### Luminex assays

For the rhesus macaques that received intravenous delivery of liposomes, maternal peripheral blood immunomodulatory proteins and growth factors were analyzed with a Cytokine/Chemokine/Growth Factor 37-plex NHP ProcartaPlex kit (ThermoFisher Scientific, cat no. EPX370-40045-901). Plasma samples were run in duplicate on a Bioplex 200 instrument (Bio-Rad, cat no. 171000201) and were analyzed with the Bioplex Manager Software as previously described 22. An analyte was included for analysis if it was detected in all animals for at least one time point. If one or two values for an individual female were below the lower limit of quantification (LLOQ), the LLOQ was substituted for the sample(s) that were undetectable.

### Statistical analysis

A mixed-effects model with a post-hoc Bonferroni multiple comparisons correction was used to analyze differences in maternal CBC parameters and analytes in maternal circulation across timepoints. These data are summarized as the mean ± standard deviation at each time point and the 95% confidence interval of the mean difference between 0 h – 4 h and 0 h - 24 h. All analyses were conducted using GraphPad Prism software.

## Results

### iRGD liposomes administered into the maternal circulation selectively target the placenta

The iRGD liposome formulations (n = 10) used in this study had a mean Z-diameter of 186 ± 4.1 nm and a mean polydispersity index (PDI) of 0.1±0.01. The ARA liposome formulations (n = 3) had a mean Z-diameter of 178 ± 4.7 nm and a mean PDI of 0.07 ± 0.03; these parameters are in line with the iRGD and ARA-decorated liposome formulations used in our previous studies 15-17, 23, where their stability and release profiles have previously been defined. Initial feasibility studies were performed to deliver iRGD liposomes via intraplacental injection or intravenous infusion into three nonhuman primate species. Liposomes delivered by intravenous infusion to a marmoset (Figure S1, A-I) and a cynomolgus macaque (Figure S1, J-Q) exhibited a widespread signal throughout both placental discs, which was generally localized to the placental syncytiotrophoblast and not observed within the underlying villous mesenchyme. In contrast, liposomes delivered by ultrasound-guided intraplacental injection into a pregnant rhesus macaque (Figure S1, R-AA) resulted in discrete areas of fluorescent signal not clearly localized to the syncytiotrophoblast (Figure S1, R, T), with many areas of the placental discs lacking a detectable signal (Figure S1, R, U). Thus, subsequent experiments utilized intravenous administration in rhesus macaques to achieve broader distribution of liposomes throughout the placenta and to evaluate targeting specificity. The demographics for each pregnancy including gestational age at infusion, fetal sex, and fetal and placental weights at 24 h post-infusion is provided in Table S1.

**Figure 2:**
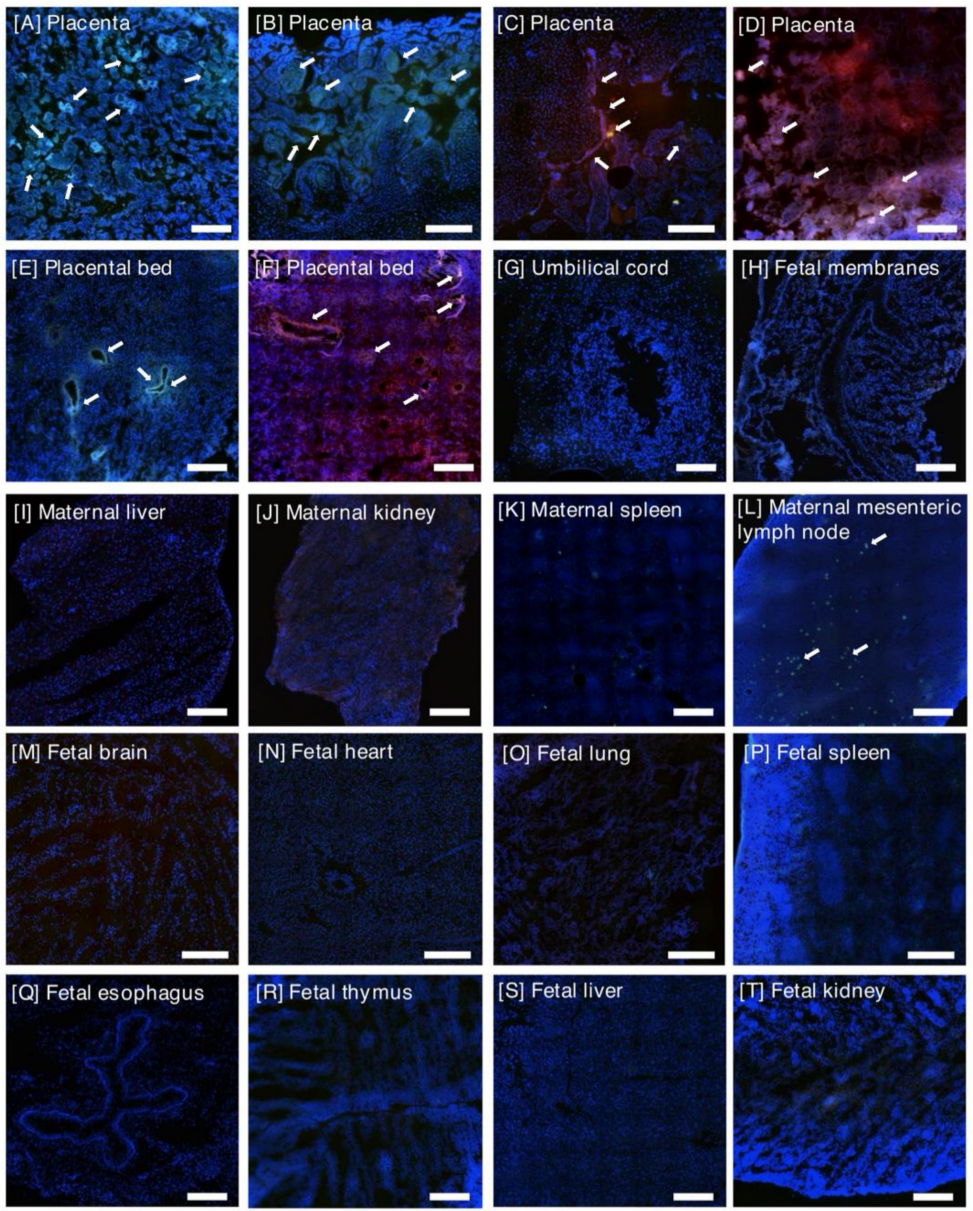
** Representative images of tissues collected from pregnant rhesus macaques, 24 h after an intravenous infusion of iRGD liposomes (n = 8). [A-D]** Placenta, **[E-F]** Placental bed, **[G]** Umbilical cord, **[H]** Fetal Membranes,** [I]** Maternal Liver, **[J]** Maternal Kidney, **[K]** Maternal Spleen, **[L]** Maternal Mesenteric Lymph Node, **[M]** Fetal brain, **[N]** Fetal heart, **[O]** Fetal lung, **[P]** Fetal spleen, **[Q]** Fetal esophagus, **[R]** Fetal thymus, **[S]** Fetal liver, **[T]** Fetal kidney. iRGD targeting peptide (rhodamine; red); liposome cargo (FAM; green); rhodamine and FAM colocalization (green-yellow; yellow; red-yellow). DAPI-positive nuclei (blue). Arrows indicate co-localization of FAM and rhodamine. Scale bar = 100μm.

Infusion of iRGD-decorated liposomes resulted in robust fluorescent signal throughout both placental discs after 24 h, which predominantly localized to the cytoplasm of cells within placental villi and the vasculature of the placental bed (Figure 2; Figure S2). Areas of red (peptide) and green (FAM cargo) fluorescence were evident, with numerous areas of colocalization present. As the FAM fluorophore self-quenches when encapsulated within the liposomes, evidence of green fluorescence is indicative of cargo release. Also, given that the relative amounts of peptide and FAM may differ slightly within the same region of tissue, colocalization may appear as yellow-green regions (more FAM than peptide), yellow regions (similar levels of peptide and FAM) and red-yellow regions (more peptide than FAM). Of the eight iRGD-infused pregnant animals studied, a fluorescent signal was evident within 75-100% of the placental biopsies examined within each animal. Fluorescence was also evident within the uterine decidua/placental bed, which was predominantly associated with the vasculature (Figure 2E, F). A weak, diffuse fluorescent signal was observed in the fetal membranes of 2 animals (Figure 2H); no signal was observed in the umbilical cord vasculature (Figure 2G). Tissues from the early gestation animal (e11) which received iRGD-decorated liposomes containing PBS exhibited a similar intensity and distribution of peptide fluorescence to all other animal receiving iRGD-decorated, FAM-loaded liposomes.

**Figure 3:**
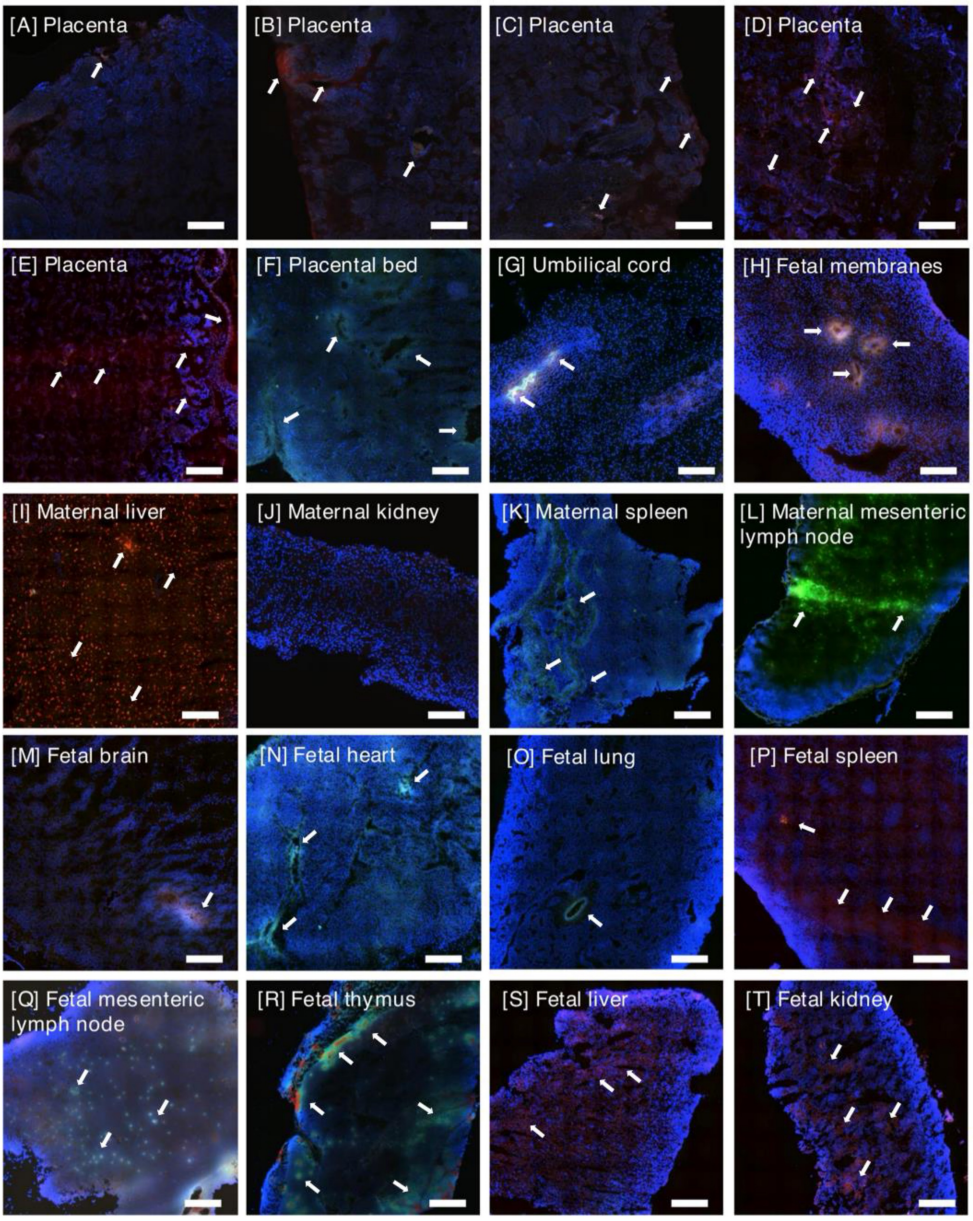
** Representative images of tissues collected from pregnant rhesus macaques, 24 h after an intravenous infusion of ARA liposomes (n=3). [A-E]** Placenta, **[F]** Placental bed, **[G]** Umbilical cord, **[H]** Fetal Membranes, **[I]** Maternal Liver, **[J]** Maternal Kidney, **[K]** Maternal Spleen, **[L]** Maternal Mesenteric Lymph Node, **[M]** Fetal brain, **[N]** Fetal heart, **[O]** Fetal lung, **[P]** Fetal spleen, **[Q]** Fetal mesenteric lymph node, **[R]** Fetal thymus, **[S]** Fetal liver, **[T]** Fetal kidney. ARA targeting peptide (rhodamine; red); liposome cargo (FAM; green); rhodamine and FAM colocalization (green-yellow; yellow; red-yellow). DAPI-positive nuclei (blue). Arrows indicate co-localization of FAM and rhodamine. Scale bar = 100μm.

**Figure 4:**
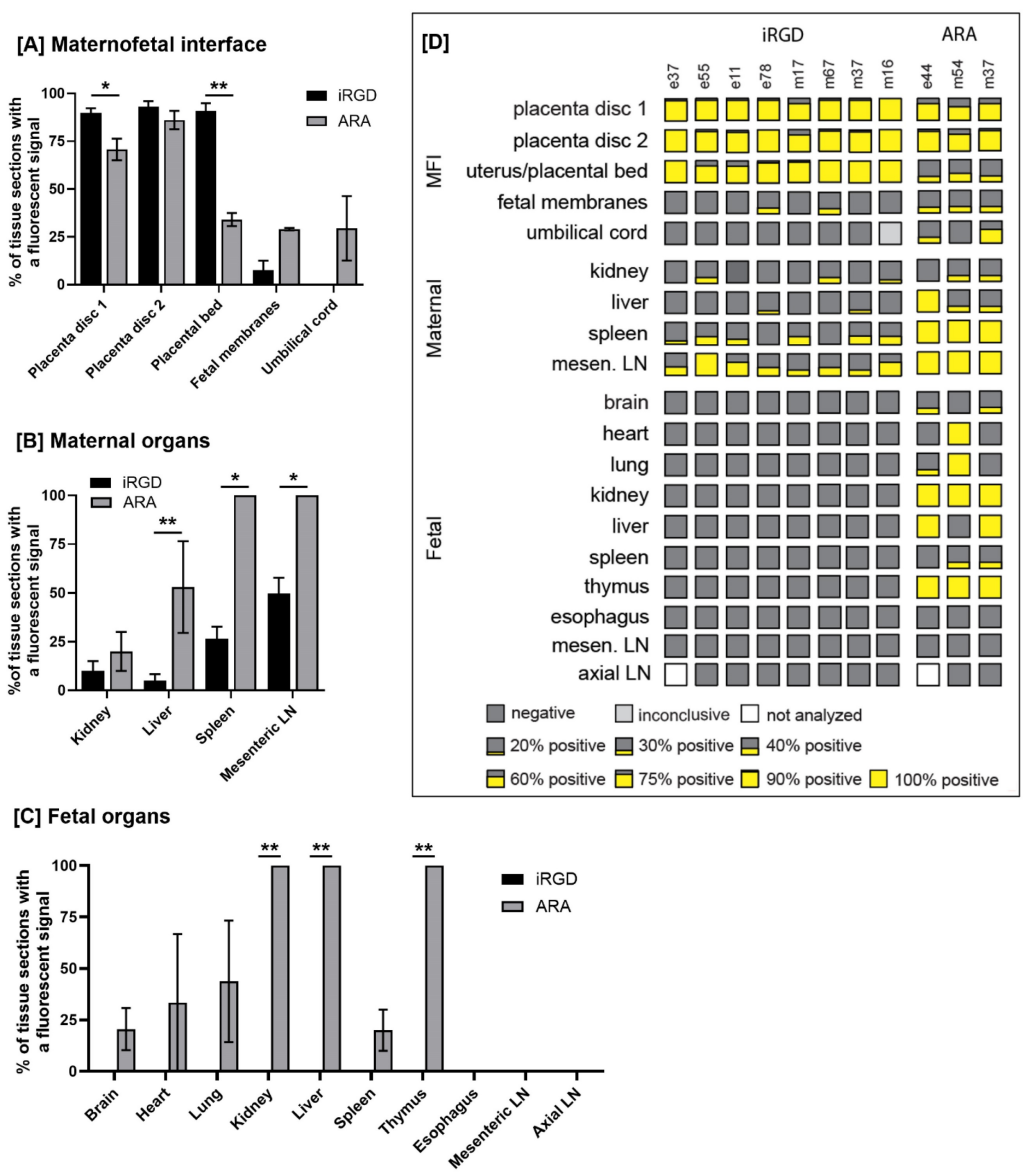
** The biodistribution of liposomes across tissue from different animals.** Maternal and fetal organs (n = 8-25 sections per organ biopsy per animal) and placental discs (n = 52–128 sections per biopsy per animal) were analyzed. To calculate the percentage of positive tissue sections per organ biopsy, the number of positive tissue sections showing evidence of a fluorescent signal was divided by the total number of tissue sections analyzed of that biopsy and multiplied by 100. The mean and standard deviation of the percentage of tissue sections with a fluorescent signal for iRGD and ARA liposomes are shown for the maternofetal interface **[A]**, maternal organs **[B]**, and fetal organs **[C]**. To prepare the fluorescence distribution map shown in panel **[D]**, the percentages of tissues sections with a fluorescent signal were rounded to the nearest 5%. MFI – maternofetal interface; LN – lymph node; e = early gestation animal; m = mid gestation animal.

Animals perfused with ARA-decorated liposomes also exhibited a fluorescent signal throughout both placental discs, indicative of placental liposome accumulation; however, the signal was weaker and was present in only 60-75% of biopsies examined (Figure 3). Limited fluorescence was associated with the placental bed vasculature, but this was weaker than that observed in animals receiving iRGD-decorated liposomes. Accumulation of ARA liposomes was also present in some areas of the fetal membranes (Figure 3G) and in the umbilical vascular endothelium (Figure 3F). The biodistribution of liposomes across tissue from different animals is summarized and quantified in Figure 4. A greater number of placental and placental bed tissues from animals receiving iRGD liposomes showed evidence of a fluorescent signal, indicative of liposomal accumulation and cargo release, than from animals receiving ARA liposomes (Figure 4A). Conversely, significantly more tissue sections of maternal liver, kidney and spleen, and fetal kidney, liver and thymus from animals receiving ARA liposomes showed evidence of a fluorescent signal, indicative of significant off-target delivery (Figure 4B, C).

### Off-target iRGD liposome accumulation is restricted to the maternal clearance organs

In the maternal compartment, a weak, diffuse fluorescent signal was observed in the kidney (n = 3 animals; 20-30% of examined samples positive), liver (n = 2 animals; 20% of samples positive) and spleen (n = 6 animals; 20-40% of samples positive), 24 h after infusion of iRGD-decorated liposomes (Figures 2, 4). A diffuse, punctate signal was also observed in the mesenteric lymph nodes of every animal, with 30-100% of samples examined positive for fluorescence (Figures 3, 4). This is consistent with the detection and removal of a proportion of the liposomes by the maternal clearance organs. As these were survival surgeries, only a limited number of maternal organs (i.e., liver, spleen, kidney and lymph node) could be biopsied for assessment. In contrast, a comprehensive analysis of fetal tissues was performed, and no evidence of iRGD peptide or FAM fluorescence was noted in any tissue examined. In contrast, after infusion of ARA-decorated liposomes, 100% of all maternal spleen and mesenteric lymph node samples assessed exhibited evidence of a fluorescent signal (Figure 3K, L). Two animals exhibited liposomal accumulation in the kidney (30% of samples positive) and all three animals exhibited liposomal accumulation in the liver (30-100% of samples positive; Figure 3I, J). The maternal liver exhibited a greater fluorescent signal from the ARA peptide than the FAM cargo, whereas a greater signal from the FAM cargo was observed in the maternal spleen and mesenteric lymph node. In the fetuses, a fluorescent signal was detected in the brain, heart, lung, kidney, liver, spleen and thymus of different animals, with only the esophagus, mesenteric lymph nodes and axial lymph nodes testing negative (Figures 3, 4). Signal from the ARA peptide was predominantly seen in the fetal liver, kidney and spleen whereas the FAM cargo was predominantly seen in the fetal heart, lung, mesenteric lymph node and thymus. These data demonstrate that targeted iRGD liposomes are more effective mediators of uteroplacental-specific payload delivery and can be used to minimize fetal drug delivery. Conversely, non-targeted ARA-decorated liposomes accumulated within the placenta but were also observed within all maternal tissues examined. A significant quantity of ARA-decorated liposomes was also transferred to the fetal compartment, resulting in a wide biodistribution and robust fluorescent signal throughout the majority of fetal organs examined.

### Maternal and fetal responses to liposome infusion were minimal

To monitor fetal response and pregnancy health, fetal heart rate was measured by ultrasound. All fetal heart rate measures throughout the duration of the study were within range for gestational age 19 or were slightly elevated (Figure S3). None of the observed fetal heart rate measures were of clinical concern, suggesting that fetal health was not impacted within 24 h following infusion.

**Figure 5:**
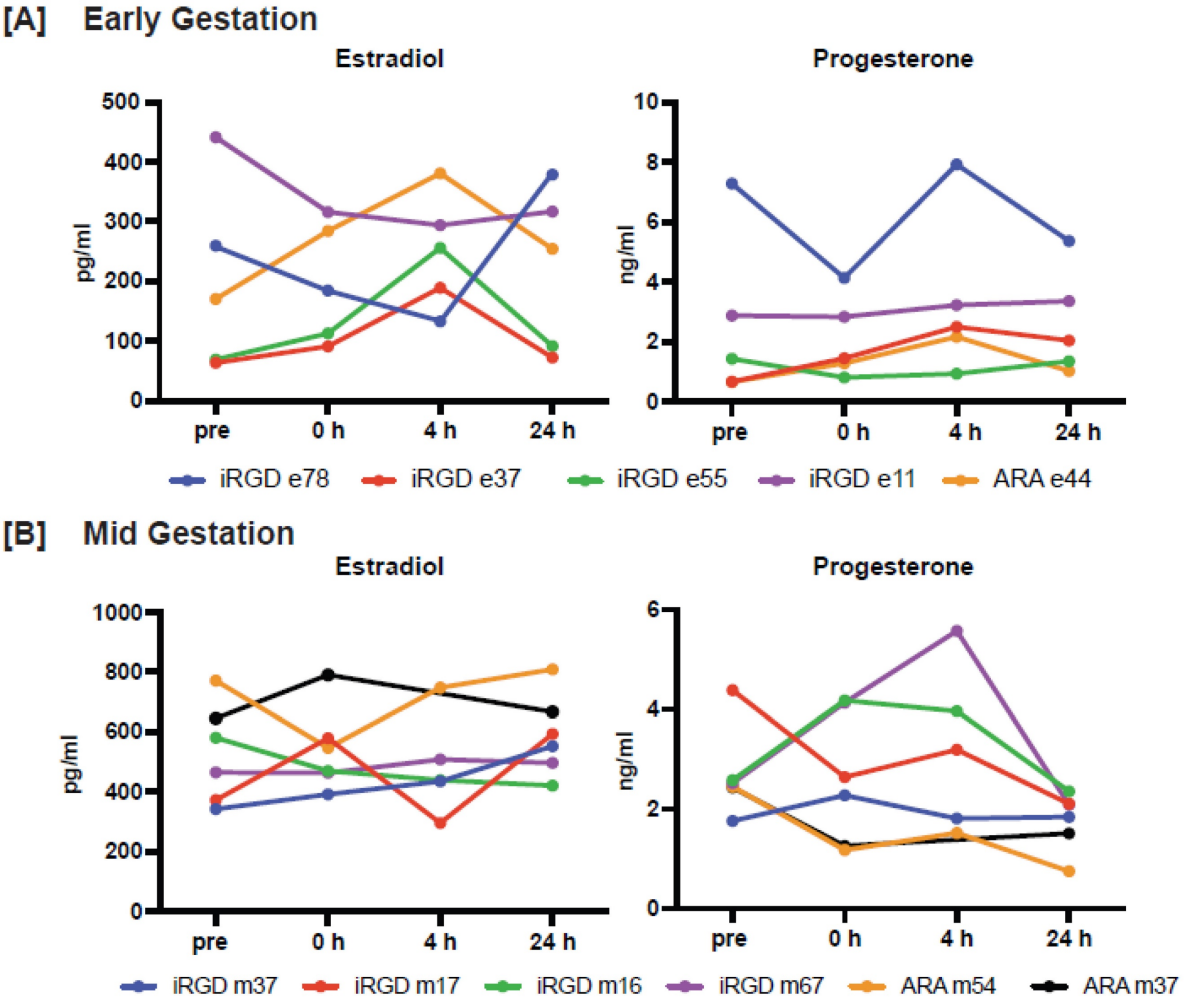
** Estradiol and progesterone levels in the maternal circulation.** Hormone levels are shown for each time point (pre, ~ 1 week prior to treatment; 0 h immediately prior to pre-treatment; and 4 and 24 h post-treatment) and animals treated in **[A]** early gestation (n = 4 iRGD, n = 1 ARA) and **[B]** mid gestation (n = 4 iRGD, n = 2 ARA).

Maternal blood samples were evaluated for levels of pregnancy associated hormones (i.e., estradiol and progesterone), CBC parameters, and for inflammatory responses. The levels of circulating estradiol and progesterone varied across females; however, an individual’s level of each hormone was relatively consistent throughout the duration of the study and did not appear to be impacted by liposome infusion (Figure 5, Table S2). Indeed, hormone values were within normal range for rhesus macaques at the respective gestational ages (personal communication, Dr. Amita Kapoor, WNPRC Assay Services Unit; estradiol: GD 45-64 mean 181.8 pg/mL ± 102 SD, range 59.0-493.0 pg/mL, n = 25; GD 84-100 mean 456.3 pg/mL ± 169.9 SD, range 244.0-760.0 pg/mL, n = 17; progesterone: GD 45-64 mean 1.36 ng/mL ± 1.0 SD, range 0.31-5.27, n = 25; GD 84-100 mean 3.0 ng/mL ± 1.0 SD, range 1.4-5.0 ng/mL, n = 17).

**Figure 6:**
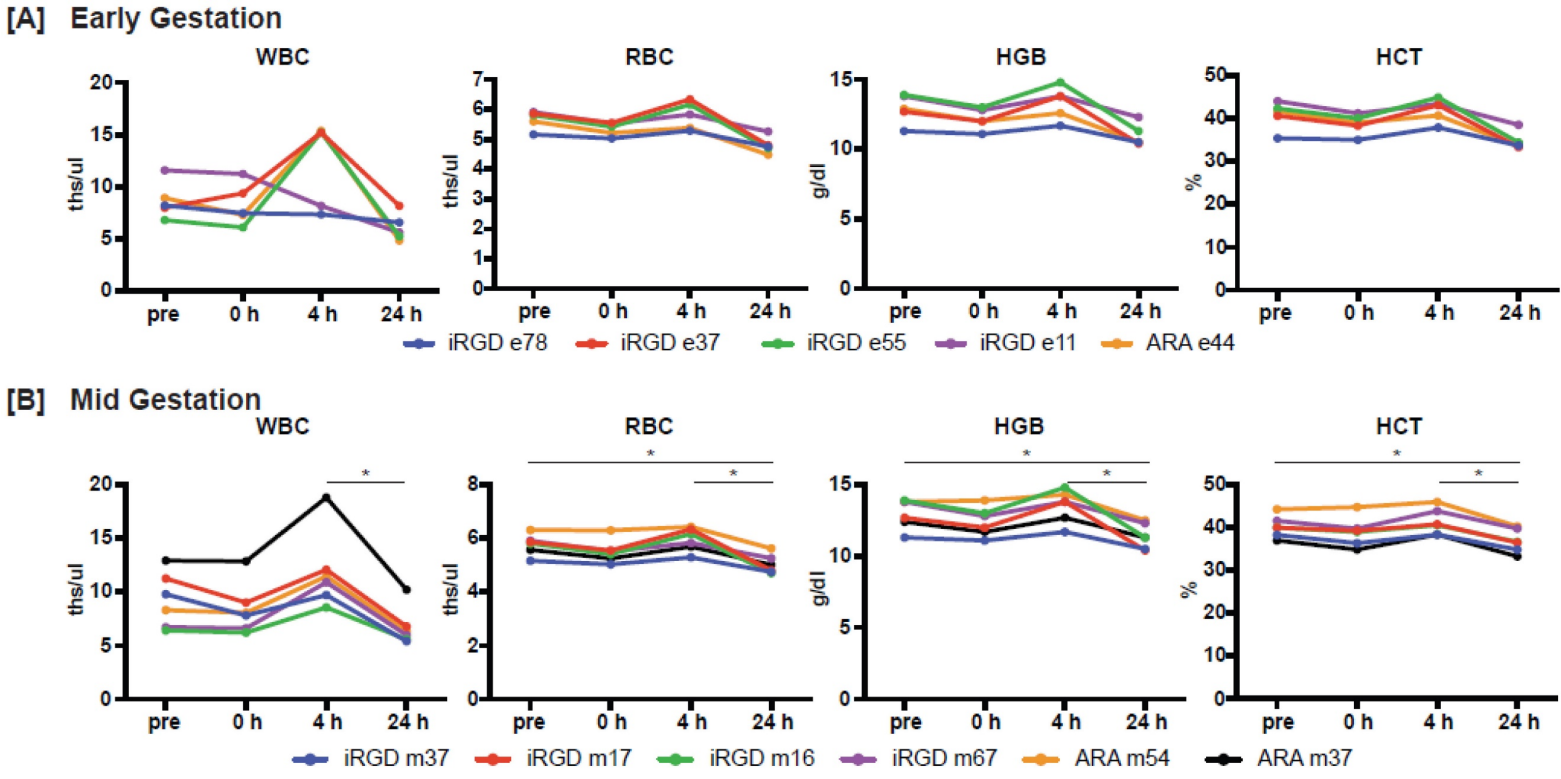
** Maternal complete blood cell counts.** Data for each animal treated in **[A]** Early gestation (n = 4 iRGD, n = 1 ARA) and **[B]** Mid-gestation (n = 4 iRGD, n = 2 ARA) at pre (~ 1 week prior to treatment), 0 h (pre-treatment), and 4 and 24 h post-treatment. Asterisk denotes an adjusted p-value of <0.05 Abbreviations: white blood cells (WBC), red blood cells (RBC), hemoglobin (HGB), hematocrit (HCT).

In terms of CBC parameters, the levels of red blood cells (RBC), hemoglobin (HGB), and hematocrit (HCT) were significantly different across timepoints with evidence of elevation in some animals at 4 h post-infusion in early pregnancy and return to baseline values; pairwise comparisons with correction for multiple testing were not significant (Figure 6, Table S3). In contrast, white blood cells (WBC), RBC, HGB and HCT were significantly elevated at 4 h post-infusion compared to 24 h post-infusion in females that received iRGD-decorated liposomes in mid-gestation, however, no differences were observed between 0 h and 24 h post-infusion suggesting that values returned to baseline (Figure 6, Table S3). The levels of RBC, HGB and HCT in mid-gestation iRGD-infused females were significantly lower at 24 h versus the pre study timepoint (~ 1 week prior) which could be attributed to the frequency of blood being withdrawn during the one-week study period.

**Figure 7:**
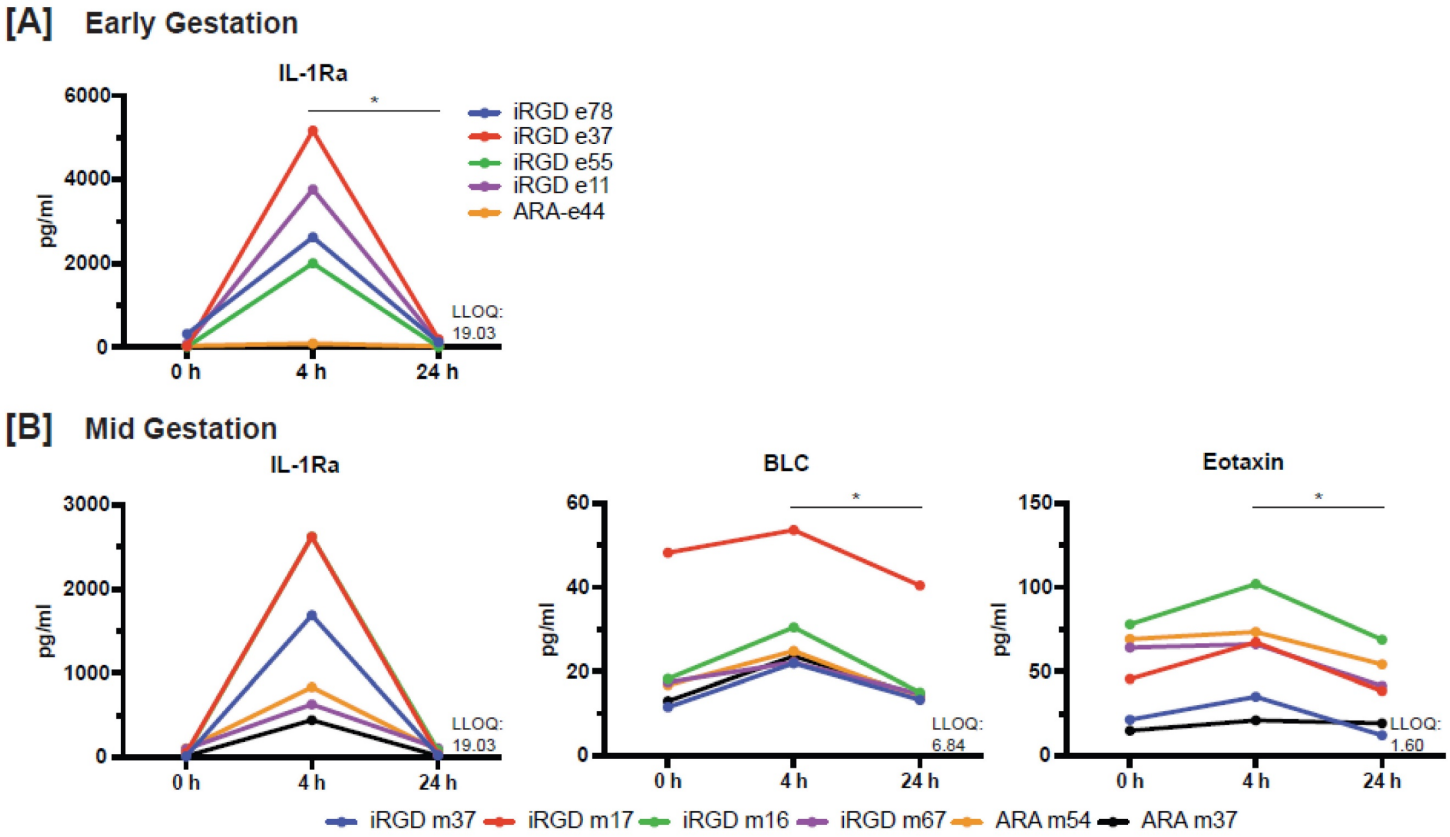
** Levels of cytokines, chemokines and growth factors in the maternal circulation. [A]** Levels of IL-1Ra in early gestation (n = 4 iRGD, n = 1 ARA). **[B]** Levels of IL-1Ra, BLC, and eotaxin in mid-gestation (n = 4 iRGD, n = 2 ARA). Asterisk denotes an adjusted p-value of <0.05.

Levels of cytokines, chemokines and growth factors in the maternal circulation were relatively unchanged throughout the study period. A total of eight analytes were consistently detected in maternal blood in both early and mid-gestation including B lymphocyte chemoattractant (BLC; alias CXCL13), eotaxin, interleukin-1 receptor antagonist (IL-1Ra), stem cell factor (SCF), IL-8, monocyte chemoattractant protein-1 (MCP-1), platelet-derived growth factor-BB (PDGF-BB) and stromal cell-derived factor-1a (SDF-1a), whereas other analytes were undetectable or only detected in a few animals. In animals that received iRGD liposomes in both early and mid-gestation, the levels of IL-1Ra were significantly different across timepoints with elevation at 4 h post-infusion, however, levels declined back to baseline by 24 h post-infusion; pairwise comparisons with correction for multiple testing were not significant in mid-gestation (Figure 7, Table S4). The levels of BLC and eotaxin were significantly elevated at 4 h in animals infused in mid-gestation and the levels of BLC also returned to baseline by 24 h post-infusion (Figure 7, Table S4). Of note, macrophage inflammatory protein-1α (MIP-1α) and MIP-1β were detectable at 4 h post-infusion for all but one animal, whereas levels were predominantly below the LLOQ at 0 h and 24 h (not shown).

### Histopathological changes were minimal in animals receiving liposome infusions

In general, histopathological analysis of the maternofetal interface tissues from liposome infused pregnancies identified pathologies that were also observed in non-contemporary gestational age-matched untreated control tissues (Table S5). In a few cases, an observed pathology was likely to have been initiated prior to liposome infusion, although the precise timing of lesion onset is difficult to interpret relative to the short study period. The following abnormal histopathological features were observed in individual early gestation iRGD liposome infused pregnancies and included: thrombosed interplacental collateral vessels, transmural parenchymal ischemia of the placenta, mild neutrophilic deciduitis, and mild focal neutrophilic vasculitis within the decidua basalis. The presence of mineralization in the latter case suggests a chronic tissue change that developed prior to liposome infusion. In an early pregnancy iRGD infused pregnancy, thrombosed interplacental collateral vessels in which there was evidence of recanalization and endothelial cell migration into the organized thrombus, suggestive that this was a pre-existing histologic lesion. Similarly, in a mid-gestation iRGD liposome infused pregnancy, the placenta showed evidence of maternal vascular malperfusion and transmural parenchymal coagulative necrosis that likely occurred prior to liposome infusion.

In general, a range of histological abnormalities were observed in randomly selected, gestationally-age matched placentas from healthy pregnancies (Table S5). This highlights that a wide range of placental features deemed “abnormal” are also observed in the placentas of healthy pregnant NHPs and these abnormalities are compatible with normal placental function and pregnancy outcomes. The onset of several of the observed pathological features likely occurred well before the 24 h study period, and in addition, the increased incidence of pathological features in untreated pregnant animals strongly implies that these lesions occur independently of liposomal infusion. Notably, all fetuses appeared healthy (e.g., normal heart rates by ultrasound) and were appropriately sized for their gestational age, and had no gross or histologic abnormalities, indicating that placental function was not obviously impaired within 24 h of treatment.

One animal infused with iRGD liposomes in mid-gestation (m37) demonstrated a strong antigenic response, however, it is unclear whether this was caused by a response to the liposome infusion, a bacterial infection, or another infectious etiology not apparent by light microscopy within the tissue sections examined. This placenta exhibited multifocal acute neutrophilic intervillositis and belonged to the animal which received three liposome preparations in subsequent pregnancies and exhibited placental pathologies in each pregnancy (iRGD m37 – antigenic response and neutrophilic intervillositis; iRGD e37 - mild neutrophilic deciduitis and vasculitis, ARA m37 – multifocal intervillositis, villitis, and transmural villous ischemia). No other animals had evidence of acute antigenic responses to liposome infusions regardless of gestational age or liposome preparation and no abnormal pathologies were observed in any fetal tissues.

## Discussion

To establish the feasibility, efficacy and safety of nanoparticles for the delivery of placental therapies, pregnant rhesus macaques were infused with placenta-targeting liposomes in early and mid-gestation. Following treatment, maternal, fetal and MFI tissues were evaluated for binding and uptake of particles and delivery of liposomal cargo. Data from our initial pilot experiments indicated that systemic liposomal infusion resulted in superior targeting, when compared to intra-placental injection, so all subsequent experiments were performed using this route of administration. Our data demonstrate that in acute experiments, there was no adverse impact of liposome infusion on maternal health or fetal well-being, and there were only sporadic observations of histologic placental pathologies that were unlikely to be related to a 24 h period of exposure to the liposomes. Infusion of iRGD-decorated liposomes resulted in a detectable and consistent signal from the delivery of the FAM cargo throughout large areas of the placental syncytiotrophoblast, but not in other maternal, fetal, or MFI tissues, demonstrating the specificity of placental targeting in this nonhuman primate model. Furthermore, the syncytial localization of the payload was identical to that observed when human placental explants are cultured with the same formulation 15, 16. Hence, these results support the feasibility of the use of targeted liposomes to deliver therapeutic materials *in vivo* to the human placenta.

Over recent years, there has been a growing interest in using nanoparticles to treat common pregnancy complications such as PE and FGR 9, 24. A range of delivery approaches are being developed, including the use of polymeric nanoparticles, dendrimers, adenoviral vectors, liposomes and solid lipid nanoparticles and there is now robust evidence demonstrating that nanoparticle interventions can improve placental function and pregnancy outcomes in mice, rats, guinea pigs and human tissue culture models 9, 24. However, the majority of published studies have used non-targeted nanoparticles for placental delivery, instead relying on systemic administration and passive placental accumulation 25-30 or ultrasound-guided injection into the placenta 31-33 or uterine artery 34-36. Targeted approaches to date have been limited and include a “placenta-trophic” lipid nanoparticle 26, composed of lipid that is preferentially endocytosed by the placenta, or decoration of nanoparticles with ligands that bind to receptors overexpressed by, but not unique to the placenta 28, 37, 38. Moreover, all *in vivo* studies of systemically administered nanoparticles are still confounded by significant off-target accumulation in the maternal liver, spleen and/or fetal tissues 26-30, 37. This is in contrast to our approach, where iterative screening with a phage library was used to identify random peptides that only bind to the placental surface, resulting in far lower levels in the maternal clearance organs and no evidence of transfer to the fetuses 15, 16.

To date, there have only been two reports documenting nanoparticle administration to pregnant nonhuman primates; these studies involved local administration of a HPMA-DMEAMA co-polymer containing an insulin-like growth factor-I (IGF-I) transgene to rhesus macaques, either via direct placental injection 39 or via infusion into the uterine artery 40. The authors reported successful placental uptake and transient transgene expression in the placental syncytiotrophoblast layer via both routes of administration and no evidence of fetal transfer. No adverse maternal reactions were seen and no detrimental effects on maternal blood CBCs or levels of circulating progesterone or estrogen were observed. Similar to our observations, occasional placental histopathological abnormalities were recorded but were unlikely to be related to the IGF-I nanoparticle treatment.

Our work has taken an alternative approach to achieve placenta-specific targeting: by creating a peptide decorated liposome that selectively homes to the placenta when infused via the saphenous vein. The need for comparatively risky procedures (placental injection) and specialist clinical staff and imaging equipment to administer the formulation (uterine artery infusion) are removed. Furthermore, a wide range of bioactive agents can be encapsulated inside the liposomes, offering the possibility of targeting multiple aspects of placental function using one formulation, or combining multiple formulations to create a personalized approach to treating placental function. Our liposomes represent a flexible platform which can be adapted to meet the varying needs of pregnant individuals experiencing complications.

It is noteworthy that a weak fluorescent signal was observed in the fetal membranes of several animals receiving both iRGD- and ARA-decorated liposomes. The fetal membranes in mice, humans and non-human primates are a multi-layered tissue primarily made of two layers, the amnion and the chorion 41. The amnion is composed of epithelial cells, and the chorion is composed of chorion trophoblast cells derived from the placenta, and mesenchymal cells. Given the significant uptake of both liposome formulations into the placenta, it is not unexpected to see modest accumulation or transfer into the membranes. Whilst they are not highly vascularized, the membranes are directly connected to the placenta and are partially composed of a similar trophoblast lineage; we have also reported this phenomenon in pregnant mice 16.

In this study, ARA-decorated liposomes were used as the negative control formulation, as the ARA peptide does not preferentially bind to placental tissue *in vitro* or *in vivo* 16. In comparison, the iRGD peptide was chosen for its placental selectivity and lack of fetal transfer in mice. Thus, iRGD decorated liposomes selectively accumulate in the NHP placenta, whereas ARA-decorated liposomes passively accumulate in the maternal clearance organs, were passively endocytosed by the placenta and subsequently crossed to the fetus. The primary role of the placenta is to extract nutrients, including lipids, from the maternal plasma and transfer them to the fetus to support growth and development 42. Thus, it is to be expected that intravenous administration of a lipid bolus (the liposomes) results in efficient fetal transfer. We hypothesize that control ARA liposomes, which are designed not to bind to any specific receptors on the placental surface are internalized by classical endocytosis mechanisms, which results in efficient transfer to the fetal circulation. Undecorated lipid nanoparticles, including liposomes have been shown to internalize into cells via both clathrin-dependent and clathrin-independent endocytosis mechanisms, such as micropinocytosis. Most of these particles are endocytosed by macropinocytosis; however, clathrin-mediated endocytosis is a prerequisite 43, 44. Conversely, we and others have proven that iRGD-decorated nanoparticles are internalized following binding to alphav integrin and neuropilin-1, both in the placenta and in tumor cells, which bypasses classical endocytosis mechanisms and ensures the retention of the nanoparticle and its cargo in the target cell 16, 45, 46.

In non-pregnant animals, the majority of targeting and non-targeting nanoparticles show significant accumulation in the liver and spleen. This is likely because the liver has a much larger tissue volume compared to the target tissue (usually a tumor), and both the liver and spleen are more highly vascularized and contain more phagocytic cells than the target tissue. These tissues represent large sinks for passive accumulation of both targeted and non-targeted liposomes. However, in pregnant animals from mid-pregnancy onwards, the placenta(s) represents an equal or greater tissue volume and when compared to the liver, and its villous structure further increases its surface area for nutrient uptake. Thus, in pregnancy, the liver and spleen represent smaller, and less efficient sinks for passive nanoparticle capture in comparison to the placenta. This is evidenced by the very limited accumulation of the targeted iRGD liposomes in the maternal liver, and the lower-than-expected accumulation of the non-targeted ARA liposomes. These results are comparable to our previous observations in pregnant mice 15, 16, and highlight the excellent specificity of our placental-targeted liposomes.

The observation that a significant proportion of the ARA-decorated liposomes accumulated in the fetus has raised the possibility that this formulation could be used for fetal drug delivery. We have previously reported in mice that intravenous administration of undecorated- and ARA-decorated liposomes with the same composition used in this study crossed the placentas of pregnant mice and accumulated in the fetuses 16. However, data from this study show non-specific accumulation of ARA-decorated liposomes in the fetal brain, heart, lung, kidney, liver, spleen and thymus, as well as significant retention in the maternal liver, spleen and mesenteric lymph nodes. Given the widespread, non-specific nature of the liposomal biodistribution in mother and fetus, this would likely reduce the efficacy of any drugs administered using this method and also limit the number of drugs deemed safe enough to be distributed across multiple fetal tissues. Thus, whilst an attractive approach in theory, additional improvements will be required for this to be a safe, effective and clinically acceptable approach in humans.

There are a series of potential safety considerations associated with liposomal infusion of PEGylated liposomes in humans 47. When introduced into the blood, a range of plasma proteins can adsorb to the liposomal surface, with different consequences. These include albumin, apolipoprotein, fibrinogen, immunoglobulins and complement 48. Binding of albumin to the nanoparticle’s surface can prevent their recognition by the mononuclear phagocytic system (MPS) and reduce their uptake and clearance. Conversely, immunoglobulins, apolipoprotein and complement components can trigger nanoparticle opsonization, which promotes their phagocytosis and clearance by the MPS and may trigger pro-inflammatory responses. Nanoparticles may also have detrimental effects on different blood cell types, leading to adverse physiological outcomes. Direct interaction of inorganic nanoparticles with the RBC membrane has been shown to cause hemolysis, which lowers the hematocrit or may cause RBC aggregation. Similarly, various cationic nanoparticle formulations have been shown to cause platelet aggregation and stimulate the neutrophil oxidative burst. In leukocytes, inorganic nanoparticles can damage the cell membrane, cause imbalances in oxidant and antioxidant processes that lead to ROS formation and DNA damage and disrupt autophagy and endo-lysosomal pathways leading to cytotoxicity 49. Nanoparticle PEGylation has also been associated with a reduced risk of blood cell interactions, including decreasing hemolysis and erythrocyte membrane damage, preventing platelet aggregation and limiting leukocyte activation and cell death 50. Our PEGylated liposomal formulation is organic and anionic in composition, and was chosen to minimize damage to maternal, fetal and placental tissues alike. Indeed, our observation that there were only modest or transient changes in maternal blood cell numbers, hormone concentrations or inflammatory cytokines confirms this to be the case.

Other adverse effects associated with liposome administration include hypersensitivity reactions, which are rare but serious events that can cause anaphylaxis; they generally occur upon first treatment, with symptoms diminishing or disappearing with subsequent treatments. In addition, production of anti-PEG antibodies in response to an initial dose of PEGylated nanoparticles is a common occurrence, which causes accelerated clearance of liposomes administered on subsequent occasions, decreasing their circulation time and efficacy 51. Interestingly, anti-PEG antibodies have been reported in up to 25% of blood donors, most of whom had never received PEGylated drugs 51. PEG exposure is now common in daily life, via cosmetics, medicines, food packaging and household products, which may impact the success of medicines containing PEG. In this study, we saw no evidence of hypersensitivity reactions or anaphylaxis in any of the animals studied. Because blood samples were taken within 24 h of the first dose of liposomes, it is unlikely that a detectable level of anti-PEG antibodies would be present in our samples. However, future studies over longer durations and incorporating a multi-dosing strategy will allow the presence of anti-PEG antibodies to be assessed and their effects on liposome clearance rate calculated. As the systemic immunological adaptations during pregnancy lead to a degree of immune quiescence 52, one would predict that the incidence of immunological reactions against liposome formulations would be lower than in the non-pregnant population. However, nanoparticle safety has not yet been investigated in pregnant individuals; this is an area which should be prioritized in the near future.

One potential limitation of our approach is the observation that the iRGD peptide also has a high affinity for tumors and has been exploited for targeted delivery of chemotherapeutics in models of breast, pancreatic and prostate cancer 23, 53-55. Thus, a requirement for liposomal treatment in pregnancy would be a negative test for the presence of malignancies, as the drugs administered to improve placental function (growth factors, vasodilators, antioxidants, anti-inflammatory agents) may promote the growth of any pre-existing tumors.

The liposome infusions were well-tolerated within the short duration of this feasibility study as fetal heart rates and analytes in maternal circulation were modestly impacted. All animals received the same diet and were housed in the same environment, and gestational age was restricted to two specific periods, however, as expected, there is evidence of normal biological variation across analytes. A limitation to interpreting analyte levels in maternal circulation is the lack of a robust reference databases for CBC and circulating immunomodulatory analytes throughout macaque pregnancy; although, CBC and hormone levels were within species-specific reference intervals developed for the specific macaque colony. There is limited knowledge of the levels and roles of circulating immunomodulatory analytes in maternal circulation during macaque pregnancy. In this study, IL-1Ra, BLC, and eotaxin were elevated at 4 h post infusion. Eotaxin is a chemokine that declines in maternal serum throughout pregnancy and its role in pregnancy is largely unknown 56. Eotaxin and IL-1Ra, an anti-inflammatory cytokine, have both been associated with gestational diabetes mellitus in human pregnancy 57, 58. BLC, or CXCL13, is a chemokine produced by monocytes, dendritic cells and lymphocytes and its role is to attract T and B lymphocytes to areas of inflammation 59, 60. Collectively, there is limited knowledge of the roles of these analytes in human and macaque pregnancy, however, the return of the values to near baseline suggests minimal impact. In addition, the baseline values were similar to our previous study at the same gestational age 39. Furthermore, similar placental histopathological features were observed in both age-matched normal and liposome infused placentas, and in addition, the manifestation of some feature(s) had likely occurred prior to liposome infusion suggesting minimal to no acute detriment to placental function.

Given the practical constraints of this pilot study, subsequent long-term safety and efficacy studies are needed fully determine the impact of liposome targeting on maternal, fetal and placental health. Future studies should include a larger cohort of pregnant macaques, non-infused control pregnancies and pregnancies infused with free FAM to assess a broader range of biological and pharmacokinetic parameters, as well as the long-term safety of the placenta-specific liposome treatment (i.e., inclusion of biomarker/analyte assessment for toxicity as well as liver and/or renal health). The enrollment of more animals and the inclusion of additional control experimental groups would bolster the statistical power to assess treatment-specific outcomes and biological variation between animals. A more detailed investigation of the targeting mechanisms involved would also support more rapid clinical translation. In this study, ARA-decorated liposomes were deemed a more rigorous negative control formulation as our previous studies in mice and human tissues showed very similar *in vitro* and *in vivo* localization as compared to non-decorated liposomes 16. Indeed, quantification of fluorophore concentrations in organ biopsies using LCMS or fluorometric assay in future studies would provide additional biodistribution and metabolism data and address whether that FAM quenched or degraded more quickly in one organ compared to another. However, given the large number of *in vivo* studies that have utilized FAM, the absence of publications on this topic suggests this is an unlikely possibility.

Rhesus macaques are an ideal model for preclinical human pregnancy studies given their similar hemochorial placental architecture, although there are some differences between nonhuman primate and human pregnancy, including shallower trophoblast invasion and the limited occurrence of adverse pregnancy outcomes such as FGR or PE in macaque pregnancy 61, 62. The lack of a naturally occurring placental insufficiency condition or comparable experimental model in the macaque makes it challenging to robustly assess the efficacy of liposome treatment prior to preclinical human studies. Macaque pregnancy studies are critical to the comprehensive evaluation of placenta-specific targeting in primate maternal and fetal tissues, and future studies may further establish the long-term safety during primate pregnancy.

## Conclusions

In summary, we have demonstrated that iRGD-decorated liposomes can be exploited for targeted placental delivery of a fluorescent payload in pregnant rhesus macaques. The formulation was well tolerated and did not cause any adverse reactions in the mother, fetus or placenta, indicating its utility for the administration of short-term treatments to alleviate placental dysfunction. The current lack of pharmaceutical investment to create effective therapies for treating the placental dysfunction underlying PE and FGR in humans is primarily due to the risk of causing adverse effects in the developing fetus. In addition, whilst the underlying pathologies such as uteroplacental vascular dysfunction, sterile inflammation, reduced nutrient transfer, altered trophoblast turnover or placental senescence are now better understood, the causes of PE and FGR are still poorly defined. A number of new nanoparticle formulations are being created for passive placental drug accumulation, the limited biodistribution studies performed to date indicate significant off-target payload delivery 26-30, 37. Thus, our well-validated targeting approach shows significant promise for the placenta-specific delivery of therapeutic payloads in human pregnancy.

## Supplementary Material

Supplementary figures and tables.

## Abbreviations

ARA: ARALPSQRSR
BLC: B lymphocyte chemoattractant
CBC: complete blood counts
CI: confidence intervals
DSPC: 1,2-distearoyl-sn-glycero-3-phosphocholine
DSPE-PEG(2000): 1,2-distearoyl-sn-glycero-3-phosphoethanolamine-N-[methoxy(polyethylene glycol)-2000] ammonium salt
DSPE-PEG(2000) maleimide: 1,2-distearoyl-sn-glycero-3-phosphoethanolamine-N-[maleimide(polyethylene glycol)-2000] ammonium salt
E2: estradiol
EGF: epidermal growth factor
eNOS^-/-^: endothelial nitric oxide synthase knockout
EVT: extravillous trophoblasts
FAM: 5(6)-carboxyfluorescein
FGR: fetal growth restriction
GD: gestational day
HCT: hematocrit
HGB: hemoglobin
IGF-I: insulin-like growth factor-I
IGF-II: insulin-like growth factor-II
IL-8: interleukin-8
IL-1Ra: interleukin-1 receptor antagonist
iRGD: CRGDKGPDC
LLOQ: lower limit of quantification
MCP-1: monocyte chemoattractant protein-1
MIP-1α: macrophage inflammatory protein-1α
MIP-1β: macrophage inflammatory protein-1β
NKG: NKGLRNK
P4: progesterone
PDGF-BB: platelet-derived growth factor-BB
PDI: polydispersity indices
PE: pre-eclampsia
RBC: red blood cells
SCF: stem cell factor
SDF-1a: stromal cell-derived factor-1a
WBC: white blood cells
WNPRC: Wisconsin National Primate Research Center
