# Supplementary material for: Targeted nanoparticles for placenta-specific drug delivery in pregnant rhesus macaques
Source: Theranostics. 2026 Jan 1;16(5):2118–35. doi: 10.7150/thno.115081 (PMC12712799; doi:10.7150/thno.115081)
Supplement: Supplementary file 1 — Supplementary figures and tables. [file thnov16p2118s1.pdf]

Supplemental Figure 1

Pregnant marmoset, IV infusion of iRGD liposomes, 24h

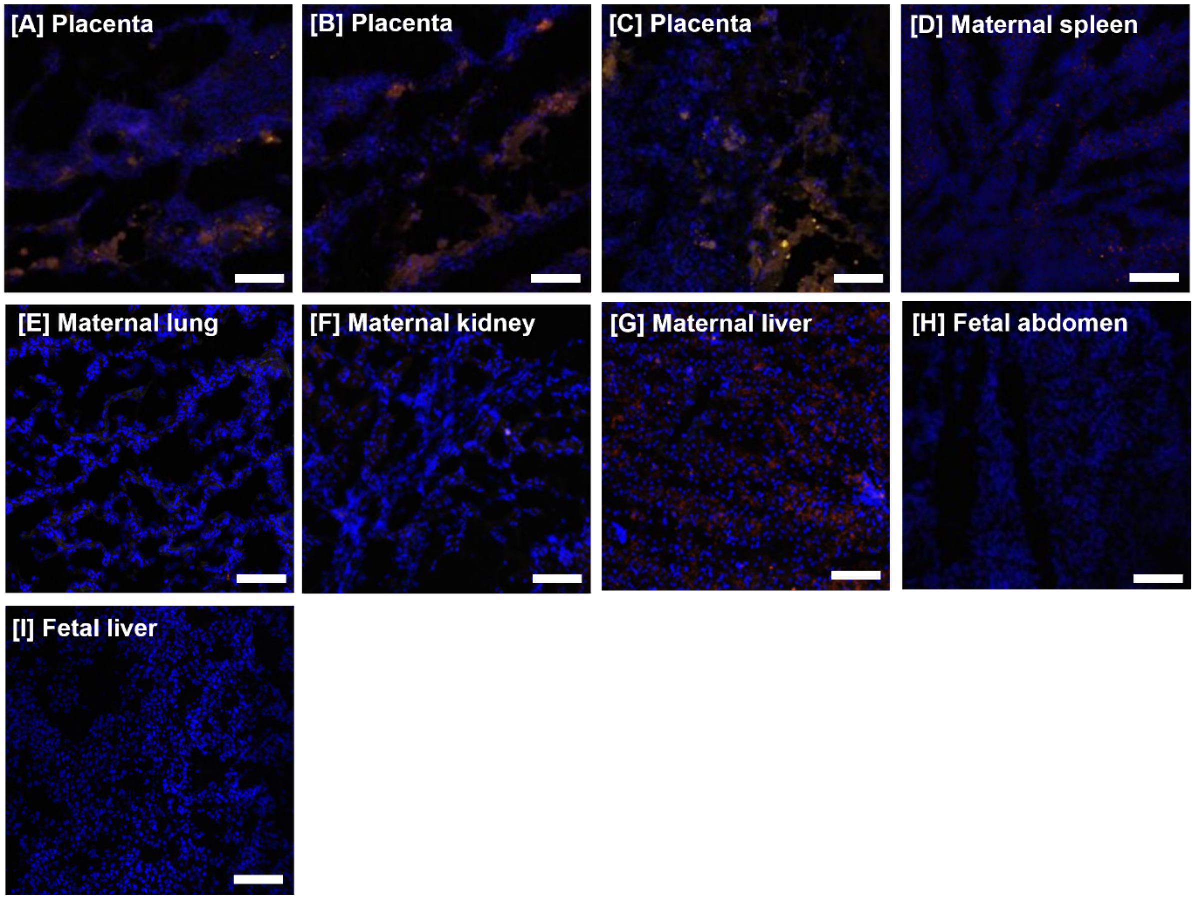

Pregnant cynomolgus macaque, IV infusion of iRGD liposomes, 24h

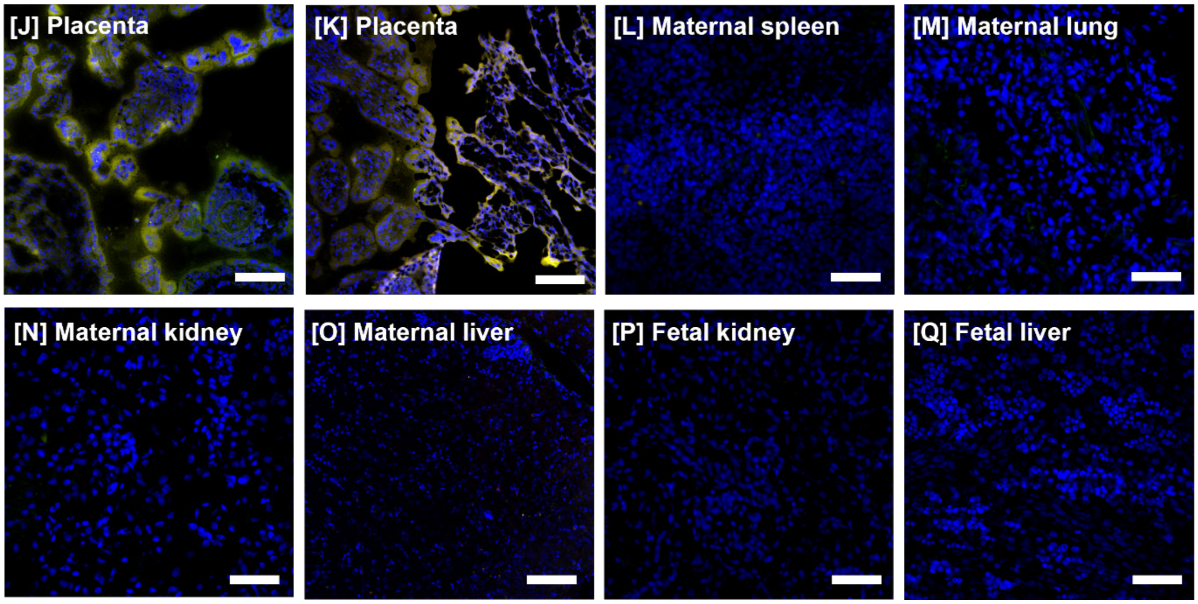

Pregnant rhesus macaque, intra-placental injection of iRGD liposomes, 24h

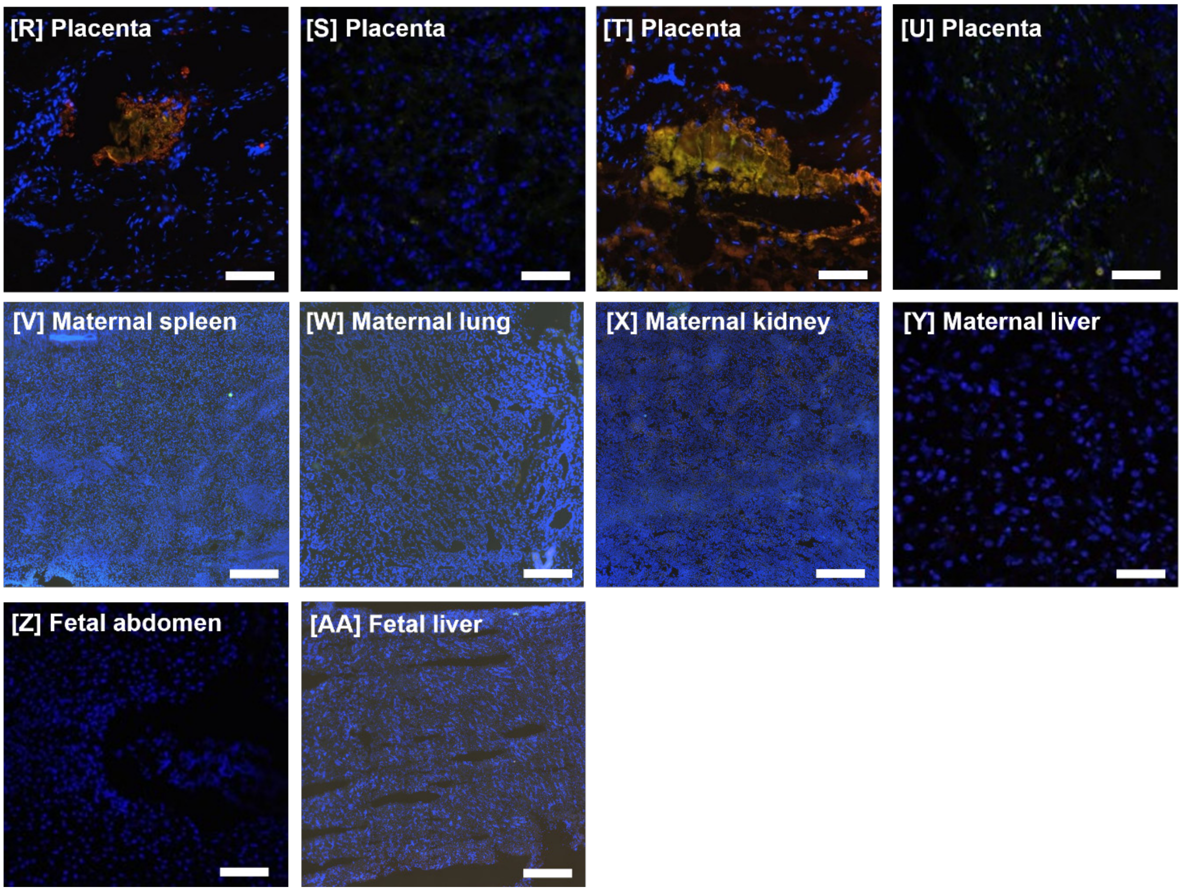

Supplemental Figure 1: Representative images of tissues collected from [A-I] a pregnant marmoset and [J-Q] a pregnant cynomolgus macaque, 24 h after an intravenous infusion of iRGD liposomes. [R-AA] Tissues collected from a rhesus macaque 24 h after an intra-placental injection of iRGD liposomes. iRGD targeting peptide (rhodamine; red); liposome cargo (FAM; green); rhodamine and FAM colocalization (green-yellow; yellow; red-yellow). DAPI-positive nuclei (blue). Scale bar = 100µm.

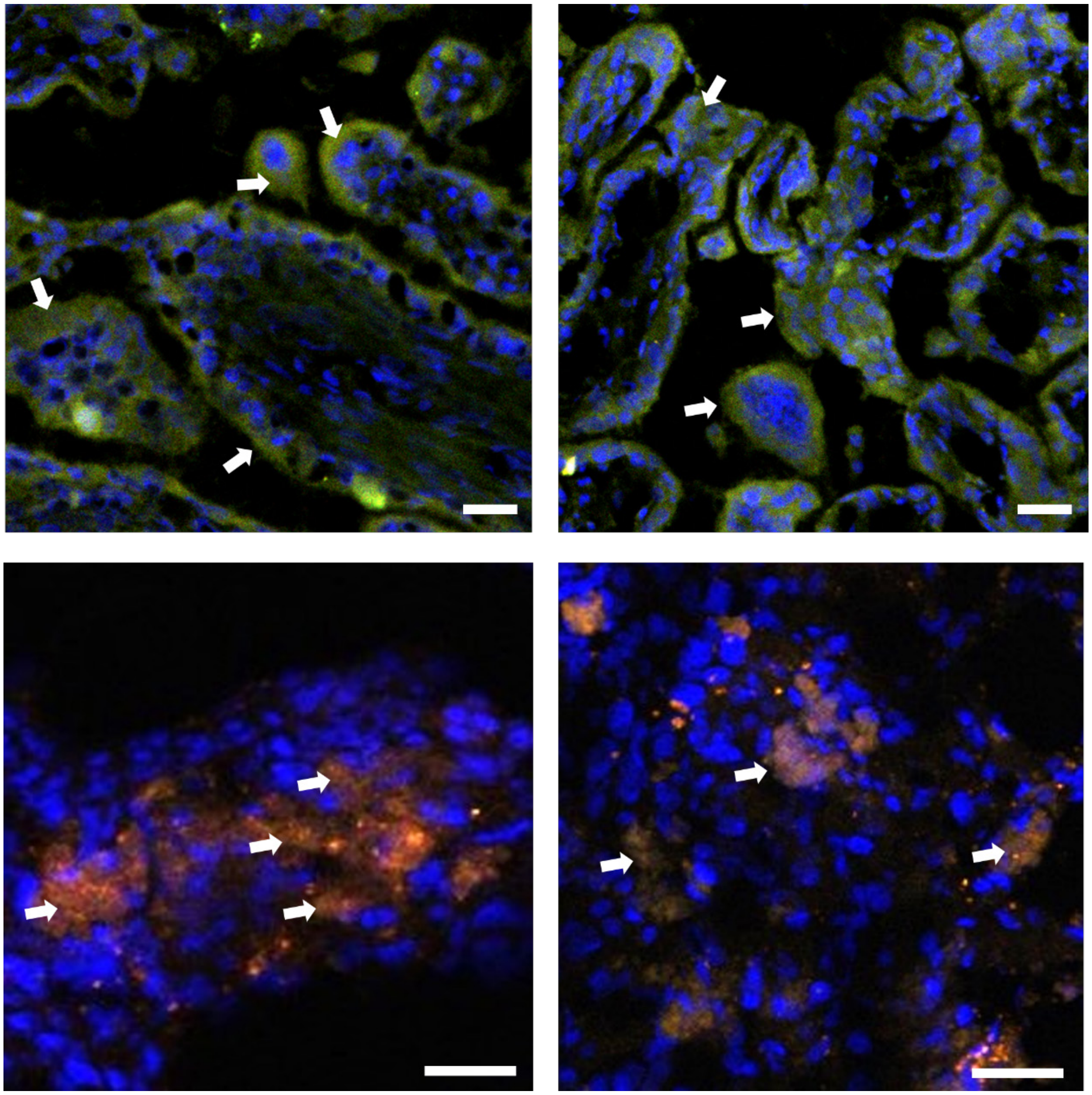

Supplemental Figure 2: Representative images of placental tissues collected from pregnant rhesus macaques, 24 h after an intravenous infusion of iRGD liposomes (n = 8). Arrows highlight cytoplasmic localization of liposomal fluorescence. iRGD targeting peptide (rhodamine; red); liposome cargo (FAM; green); rhodamine and FAM colocalization (green-yellow; yellow; red-yellow). DAPI-positive nuclei (blue). Scale bar = 25µm

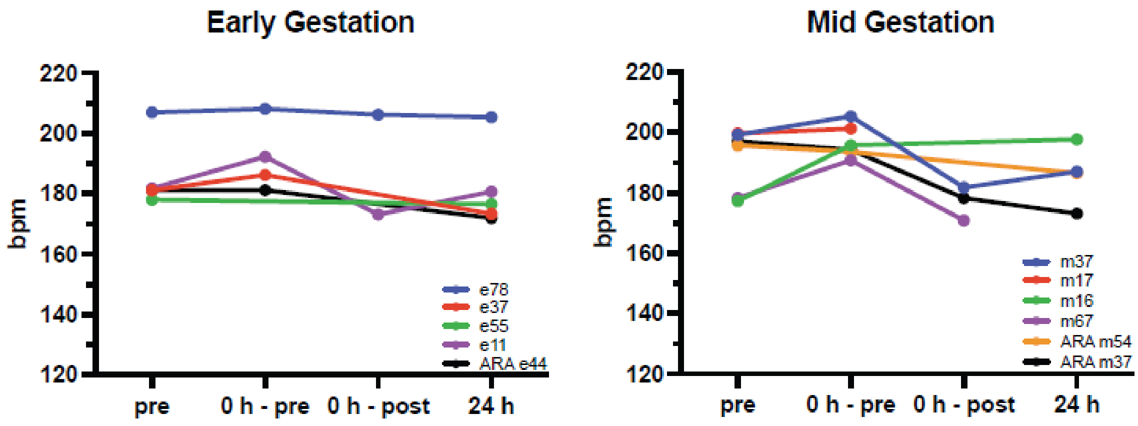

Supplemental Figure 3: Fetal heart rate measures. Heart rate in beats per min (bpm) at each time point (pre: ~ one week prior to liposome infusion, 0 h – pre: prior to liposome infusion, 0 h – post: immediately following infusion, and 24 h post-infusion) in early (n = 4 iRGD, n = 1 ARA) and mid-gestation (n = 4 iRGD, n = 2 ARA).

Supplemental Table 2. Hormone levels in the maternal circulation

		Mean ± SD				p-value	95% CI of Mean Difference	
		Pre	0 h	4 h	24 h		0 h – 4 h	0 h – 24 h
Early (n=4)	E2 (pg/ml)	208.2 ± 179.7	175.9 ± 101.4	217.7 ± 71.43	214.6. ± 155.5	0.78	-237.0 – 153.4	-255.6 – 178.2
	P4 (ng/ml)	3.1 ± 3.0	2.3 ± 1.5	3.6 ± 3.0	3.0 ± 1.8	0.33	-268.4 – 381.0	-224.2 – 144.4
Mid (n=4)	E2 (pg/ml)	439.7 ± 107.1	475.6 ± 77.4	419.3 ± 89.3	515.5 ± 74.9	0.43	-4.9 – 2.2	-1.4 – (-0.01)
	P4 (ng/ml)	2.8 ± 1.1	3.3 ± 1.0	3.6 ± 1.6	2.1 ± 0.2	0.22	-2.1 – 1.5	-0.5 – 3.0

Supplemental Table 3. Maternal complete blood count parameters

*Denotes adjusted p-value <0.05 for the 95% confidence intervals (CI) of the mean difference

		**Mean ± SD**				**p-value**	**95% CI of Mean Difference**	
		Pre	0	4 h	24 h		0 h – 4 h	0 h – 24 h
Early n=4	WBC (ths/uL)	8.7 ± 2.0	8.6 ± 2.2	11.5 ± 4.3	6.4 ± 1.3	0.19	-14.4 – 8.6	-2.7 – 7.0
	RBC (10^6/uL)	5.7 ± 0.4	5.4 ± 0.2	5.9 ± 0.5	4.9 ± 0.3	0.03	-1.1 – 0.1	-0.1 – 1.1
	HGB (g/dL)	12.9 ± 1.2	12.2 ± 0.9	13.5 ± 1.3	11.1 ± 0.9	0.02	-2.6 – (-0.04)*	-0.2 – 2.4
	HCT (%)	40.6 ± 3.7	38.6 ± 2.7	42.3 ± 3.1	35.0 ± 2.4	0.01	-6.5 – (-0.9)*	-0.5 – 7.8
	MCV (fL)	71.3 ± 2.8	71.8 ± 2.9	71.7 ± 2.7	71.7 ± 1.9	0.79	-2.9 – 2.9	-2.4 – 2.5
	MCH (pg)	22.7 ± 1.1	22.8 ± 1.0	22.9 ± 1.2	22.8 ± 1.1	0.51	-0.7 – 0.5	-0.3 – 0.2
	MCHC (g/dL)	31.9 ± 0.7	31.7 ± 0.6	31.9 ± 0.9	31.8 ± 0.8	0.65	-1.8 – 1.2	-1.3 – 1.0
	RDW (%)	13.6 ± 0.9	13.6 ± 0.9	13.9 ± 1.1	13.6 ± 0.9	0.20	-1.3 – 0.5	-0.7 – 0.5
	PLT (ths/uL)	343.8 ± 150.9	391.8 ± 91.4	310.8 ± 91.6	338.5 ± 123.2	0.55	-174.0 – 336.0	-29.7 – 136.2
	MPV (fL)	11.4 ± 1.8	11.3 ± 1.9	11.7 ± 1.3	11.3 ± 1.7	0.50	-1.9 – 1.2	-0.2 – 0.3
Mid n=4	WBC (ths/uL)	8.5 ± 2.4	7.4 ± 1.3	10.3 ± 1.5	5.9 ± 0.6	0.02	-5.1 – (-0.7)*	-0.6 – 3.5
	RBC (10^6/uL)	5.6 ± 0.3	5.3 ± 0.2	5.7 ± 0.3	5.1 ± 0.3	0.003	-0.7 – (-0.03)*	-0.1 – 0.5
	HGB (g/dL)	12.7 ± 0.4	12.1 ± 0.4	12.9 ± 0.5	11.7 ± 0.6	0.001	-1.6 – (-0.02)*	-0.4 – 1.3
	HCT (%)	40.0 ± 1.3	38.6 ± 1.5	40.9 ± 2.3	37.0 ± 2.0	0.006	-4.8 – 0.2	-0.9 – 4.2
	MCV (fL)	71.6 ± 2.9	72.5 ± 2.8	71.8 ± 2.5	72.0 ± 2.7	0.18	-0.7 – 2.0	0.2 – 0.8
	MCH (pg)	22.7 ± 0.7	22.7 ± 0.6	22.7 ± 0.7	22.7 ± 0.5	0.84	-0.7 – 0.6	-0.3 – 0.2
	MCHC (g/dL)	31.7 ± 0.4	31.3 ± 0.4	31.6 ± 0.7	31.6 ± 0.5	0.42	-1.8 – 1.3	-0.6 – 0.1
	RDW (%)	13.8 ± 0.6	14.0 ± 0.5	14.1 ± 0.5	14.0 ± 0.6	0.32	-0.3 – 0.2	-0.2 – 0.2
	PLT (ths/uL)	478.8 ± 157.3	501.0 ± 136.8	458.5 ± 184.2	424.5 ± 65.2	0.40	-99.0 – 184.0	-81.6 – 234.6
	MPV (fL)	11.3 ± 0.8	10.7 ± 0.6	11.3 ± 0.4	11.1 ± 0.8	0.50	-2.4 – 1.4	-1.0 – 0.3

Supplemental Table 4. Immunomodulatory analytes in the maternal circulation

1 = LLOQ substituted for one value; 2 = LLOQ substituted for two values

*Denotes adjusted p-value <0.05 for the 95% CI of the mean difference

Gestational Age	Analyte	Mean ± SD (pg/ml)			p-value	95% CI of Mean Difference	
		0 h	4 h	24 h		0 h – 4 h	0 h – 24 h
Early n=4	BLC[1]	19.1 ± 9.1	23.4 ± 3.6	11.8 ± 4.0	0.0945	-20.1 – 11.6	-11.6 – 26.2
	Eotaxin	44.7 ± 28.0	67.5 ± 25.1	30.4 ± 12.1	0.1075	-91.4 – 45.7	-32.4 – 61.0
	IL-1RA[2]	109.6 ± 140.8	3391 ± 1392	111.1 ± 73.2	0.0177	-6293.0 – (-269.6)*	-313.3 – 310.3
	SCF	8.1 ± 4.8	9.0 ± 4.5	7.2 ± 4.1	0.3620	-6.1 – 4.3	-5.0– 6.8
	IL-8[1]	265.0 ± 393.3	86.56 ± 52.7	17.2 ± 15.5	0.3171	-546.6 – 903.6	-563.9 –1060
	MCP-1	75.9 ± 54.1	311.9 ± 294.7	56.8 ± 10.5	0.1658	-872.0 – 400.0	-84.1 – 122.3
	PDGF-BB[1]	980.7 ± 716.0	694.5 ± 217.4	530.8 ± 508.6	0.4765	-1143.0 – 1715.0	-1570.0 – 2470.0
	SDF-1a	53.4 ± 29.6	58.6 ± 29.8	33.6 ± 23.6	0.1082	-54.8 – 44.5	-11.3 – 51.0
Mid n=4	BLC	23.9 ± 16.5	32.0 ± 14.9	20.9 ± 13.1	0.0028	-15.9 – (-0.4)*	-5.2 – 11.20
	Eotaxin	52.4 ± 24.5	67.7 ± 27.3	40.3 ± 23.1	0.0079	-36.2 – 5.5	17.9 – 37.0
	IL-1RA[1]	51.2 ± 34.8	1892.0 ± 948.0	64.2 ± 39.0	0.0145	-3864.0 – 183.4	-62.20 – 36.2
	SCF	11.5 ± 3.1	11.7 ± 6.4	8.6 ± 2.4	0.2332	-7.4 – 7.1	-0.1 – 5.9
	IL-8[1]	8.5 ± 9.6	102.8 ± 93.9	140.7 ± 255.3	0.4179	-277.5 – 88.8	-670.2 – 405.7
	MCP-1	28.8 ± 10.3	252.7 ± 273.7	27.2 ± 4.7	0.1914	-777.7 – 330.0	-10.3 – 13.6
	PDGF-BB	452.5 ± 83.2	1295.0 ± 927.2	577.1 ± 351.4	0.1607	-2858.0 – 1173.0	-892.1 – 643.0
	SDF-1a	76.9 ± 53.2	56.2 ± 28.0	64.4 ± 38.5	0.2358	-34.1 – 75.3	-28.3 – 53.2

Supplemental Table 5. Histopathological analysis of the MFI, maternal and fetal tissues

Gestational Age	Tissue	Non-infused Control	ARA Liposome	iRGD Liposome
Early	MFI	Ctrl 1 – **p**: mild chronic retroplacental hemorrhage; **d**: minimal/mild multifocal segmental necrosis<br><br>Ctrl 2 – **p**: mild multifocal chronic villitis, chronic/acute intervillositis, moderate multifocal hemorrhage, necrosis in trophoblastic shell; **d**: acute neutrophilic deciduitis<br><br>Ctrl 3 – **p**: mild multifocal acute coagulative necrosis with mild neutrophilic infiltration; **d**: minimal neutrophilic deciduitis, acute focal coagulative necrosis	e44 – **p**: mild multifocal villous mineralization; **d**: minimal multifocal coagulative necrosis	e11 – **p**: mild-moderate multifocal transmural ischemia, coagulative necrosis, acute intervillositis, intervillous hemorrhage, villous agglutination; thrombosed interplacental collateral vessels<br><br>e37 – **p**: moderate multifocal extensive basal plate hemorrhage with villous congestion; **d**: minimal multifocal acute neutrophilic deciduitis and vasculitis<br><br>e55 – **p**: minimal multifocal villous mineralization; **d**: moderate multifocal, acute/subacute hemorrhage with adjacent mild coagulative necrosis, moderate acute deciduitis<br><br>e78 – **p**: necrosis, mild multifocal acute/chronic hemorrhage
	Maternal	Ctrl 1, 2, 3 – NSL	e44 – NSL	e11, e37, e55, e78 – NSL
	Fetal	Ctrl 1, 2, 3 – NSL	e44 - NSL	e11, e37, e55, e78 – NSL
Mid	MFI	Ctrl 1 – **p**: minimal-mild multifocal thrombosis and ischemia with minimal acute neutrophilic inflammation, moderate multifocal mineralization; **fm**: mild diffuse chorioamniotic hemsiderosis<br><br>Ctrl 2 – **p**: mild neutrophilic intervillositis, villitis with syncytial knot formation, mild multifocal basal plate necrosis, hemorrhage, mineralization, mild multifocal chorionic necrosis, acute inflammation; **d**: organized hemorrhage, moderate chronic lymphoplasmacytic deciduitis<br><br>Ctrl 3 – **p**: mild-moderate multifocal coagulative lytic necrosis, villous collapse, syncytial knot formation, mild to multifocal mineralization; **d**: mild chronic deciduitis<br><br>Ctrl 4 – **p**: mild retroplacental hemorrhage, mild multifocal basal plate infarction and vascular thrombosis with minimal multifocal mineralization, chronic multifocal ischemia; **d**: mild multifocal chronic lymphoplasmacytic deciduitis; **uc**: focal acute perivascular hemorrhage; **u/pb**: mild multifocal lymphocytic endometritis	m44 – **p**: mild multifocal villous mineralization; **d**: minimal multifocal coagulative necrosis<br><br>m37 – **p**: moderate-severe transmural villous ischemia, mild-moderate multifocal acute intervillositis, mild acute/chronic villitis	m16 – **p:** moderate transmural ischemia, minimal-mild multifocal villous mineralization; **d**: acute and chronic mild-moderate hemorrhage<br><br>m17 – **p**: minimal to moderate multifocal mineralization<br><br>m37 – **p**: multifocal acute neutrophilic intervillositis, villitis with multifocal basal plate thrombosis and ischemia<br><br>m67 – **p**: mild multifocal mineralization, mild focal segmental transmural ischemia; **d**: mild multifocal persistently muscularized arteries

		Ctrl 5 – **p**: minimal multifocal mineralization, mild multifocal villous avascularization, multifocal extensive transmural ischemia; **d**: minimal - mild multifocal lymphocytic deciduitis with rare neutrophils and focal occlusive fibrin thrombi<br><br>Ctrl 6 - **p**: NSL; **d**: moderate multifocal decidual arteriopathy with multifocal vascular fibrinoid necrosis and intraluminal thrombi with perivascular edema and multifocal perivascular and interstitial lymphoplasmacytic deciduitis<br><br>Ctrl 7 – **p**: mild multifocal sub-chorionic fibrin, minimal multifocal acute subchorionitis, rare avascular villi, moderate to marked multifocal basal plate thrombosis, mild multifocal neutrophilic villitis, intervillositis; **d**: mild-moderate chronic lymphoplasmacytic deciduitis<br><br>Ctrl 8 – **p**: mild-moderate multifocal basal plate thrombosis, minimal-mild multifocal neutrophilic intervillositis, villositis, with increased intervillous fibrin and syncytial knots, mild multifocal segmental subchorionic fibrin; **d**: minimal-mild multifocal lymphocytic deciduitis with mild diffuse hemosiderosis; **fm**: rare single neutrophils; **u/pb**: mild multifocal lymphocytic perivascular myometritis with moderate diffuse necrosupperative superficial endometritis		
	Maternal	Ctrl 3, 4, 6, 8: NSL<br><br>Ctrl 1 – **s:** mild diffuse neutrophilic splenitis. **u/m**: minimal neutrophilic vasculitis<br><br>Ctrl 2 – **u**: mild multifocal vasculitis and perivasculitis<br><br>Ctrl 5 – **s**: mild diffuse neutrophilic splenitis<br><br>Ctrl 7 – **l**: minimal periportal lymphoplasmacytic hepatitis with rare neutrophils; **s**: mild diffuse neutrophilic splenitis	m44 – NSL	m16, m17, m67 – NSL<br><br>m37 – **s:** mild acute neutrophilic splenitis and sinusoidal histiocytosis
	Fetal	Ctrl 1-8: NSL	m44 - NSL	m16, m17, m37, m67 - NSL

Abbreviations: NSL – no significant lesions, p – placenta, d – decidua basalis, fm – fetal membranes, uc – umbilical cord, u/pb – uterus placental bed, u/m – uterus myometrium, s – spleen, l – liver, u – uterus, ln – lymph node
